# Green Tea Components: In Vitro and In Vivo Evidence for Their Anticancer Potential in Colon Cancer

**DOI:** 10.3390/cancers17040623

**Published:** 2025-02-13

**Authors:** Federica Randisi, Gianpaolo Perletti, Emanuela Marras, Marzia Bruna Gariboldi

**Affiliations:** Department of Biotechnology and Life Sciences (DBSV), University of Insubria, 21100 Varese, Italy; frandisi1@studenti.uninsubria.it (F.R.); gianpaolo.perletti@uninsubria.it (G.P.); emanuela.marras@uninsubria.it (E.M.)

**Keywords:** green tea, colorectal cancer, catechins, epigallocatechin-3-gallate, in vitro studies, in vitro studies, human studies

## Abstract

Green tea consumption has been implicated in various biological activities, with particular emphasis on its anticancer properties. The antineoplastic effects of green tea are primarily attributed to its rich polyphenol content, among which, epigallocatechin-3-gallate (EGCG) is recognized as the most bioactive and potent catechin, responsible for the majority of its anticancer activity. This review provides a detailed examination of the in vitro and in vivo effects of green tea components, focusing on their potential therapeutic implications in colorectal cancer. The molecular mechanisms of action and bioactive constituents of green tea are systematically discussed, alongside an evaluation of experimental evidence supporting their efficacy. Furthermore, insights into the relationship between green tea dietary intake and colorectal cancer risk are analyzed, with a particular emphasis on clinical data and findings from meta-analyses involving patients diagnosed with colon cancer. The aggregated evidence underscores the necessity for well-designed randomized controlled trials and longitudinal cohort studies to substantiate the role of green tea as a chemopreventive agent. Additionally, future investigations should prioritize determining the optimal dosages, the appropriate durations of consumption, and the potential modulatory effects of dietary or lifestyle factors on green tea’s anticancer efficacy.

## Simple Summary

Traditionally used in Asian countries, green tea is nowadays one of the most widely used beverages worldwide. Polyphenols are the primary bioactive compounds found in green tea, with catechins representing the principal constituents. Among these, epigallocatechin-3-gallate (EGCG) is the most abundant and extensively investigated catechin. The pharmacological potential of green tea polyphenols has been well-documented, encompassing antibacterial, anti-inflammatory, antioxidant, antiobesity, antiviral, and anticancer activities. Notably, anticancer properties have been observed against several malignancies, including colorectal cancer. This review aims to comprehensively examine the anticancer effects of green tea constituents in colorectal cancer through in vitro (cell-based) and in vivo (animal-based) studies, with a focus on elucidating their underlying mechanisms of action. Additionally, particular emphasis is placed on clinical studies, including meta-analyses and key investigations involving human participants.

## 1. Introduction

Colorectal cancer (CRC) represents a major global health concern, ranking as the third most frequently diagnosed malignancy and the second leading cause of cancer-related mortality worldwide [1,2,3,4]. Current projections estimate a substantial rise in CRC burden, with the annual incidence predicted to reach 3.29 million new cases and 1.66 million deaths by 2045 (Global Cancer Observatory: Cancer Tomorrow, accessed on 5 January 2025, available online at https://gco.iarc.fr/tomorrow). Furthermore, the incidence of CRC is strongly correlated with the Human Development Index (HDI), as countries with a high HDI currently exhibit rates approximately four times higher than those observed in low-HDI regions [2,3]. However, longitudinal trends indicate a marked disparity in the growth of colorectal cancer incidence between areas with differing HDI levels. Between 2022 and 2045, colorectal cancer incidence is projected to increase by 111.8% in low-HDI countries, compared to a 67.9% increase in very high-HDI countries (Global Cancer Observatory: Cancer Tomorrow, accessed on 5 January 2025, available online at https://gco.iarc.fr/tomorrow). This anticipated rise in low-HDI regions is closely linked to increased exposure to colorectal cancer risk factors, driven by the adoption of a Westernized lifestyle, a shift often accompanying socioeconomic and industrial transitions [2,4].

Colorectal cancer can be classified into three primary subtypes based on the origin of the underlying genetic alterations: sporadic, inherited, and familial [5]. Sporadic colorectal cancer constitutes approximately 70% of all cases and is characterized by mutations affecting a range of genes. The initial driver mutation often arises in the adenomatous polyposis coli (*APC*) gene, a critical tumor suppressor, promoting the formation of benign adenomas or polyps. Subsequent mutations in other genes, such as *Kristen rat sarcoma* viral oncogene homolog (*KRAS*), *p53*, and *Deleted in Colorectal Cancer* (*DCC*), further drive tumor progression [5,6]. Inherited colorectal cancer constitutes approximately 5% of cases and encompasses both polyposis and non-polyposis syndromes. Polyposis includes familial adenomatous polyposis (FAP), while non-polyposis forms are predominantly linked to deficiencies in DNA mismatch repair proteins, including MSH2, MLH1, MSH6, PMS1, and PMS2, often classified under Lynch syndrome [7,8]. Familial colorectal cancer constitutes about 25% of cases but does not typically result from known germline mutations. Instead, it arises from complex interactions between genetic predispositions (e.g., gene–gene interactions, epigenetic modifications), environmental factors, or their combined effects [7]. Studies indicate that chromosomal abnormalities and translocations affecting key signaling pathways, including *Wnt*, *mitogen-activated protein kinases/phosphatidylinositole 3 kinase* (*MAPK/PI3K*), and (*tumor growth factor-β*) *TGF-β*, along with disruptions in cellular functions such as *p53* and cell-cycle regulation, are also involved in the pathogenesis of familial colorectal cancer [5,9].

According to the World Health Organization (WHO), several factors contribute to an increased risk of developing colorectal cancer. These include aging, a family history of the pathology, hereditary conditions such as familial adenomatous polyposis (FAP), and a personal history of colorectal cancer or adenomatous polyps. In addition to these non-modifiable risk factors, various lifestyle factors play a significant role. Among these, excess body weight is particularly notable as a major risk factor [2,10]. This relationship is believed to be mediated by the proinflammatory cytokines produced by adipose tissue, chronic hyperinsulinemia, and elevated levels of insulin-like growth factor 1 (IGF-I), all of which may facilitate carcinogenesis [11]. Dietary habits have also been extensively studied in the context of colorectal cancer risk. High consumption of processed and red meats has been linked to impaired gut immune responses and increased gut inflammation, both of which may promote tumorigenesis. Conversely, a diet abundant in fiber, fruits, vegetables, dairy products, fish, beta-carotene, vitamins C, E, and D, and folate is associated with a protective effect against colorectal cancer development [12,13,14]. Lifestyle factors beyond diet also influence colorectal cancer risk. Regular physical activity and the use of aspirin or non-steroidal anti-inflammatory drugs (NSAIDs) have been shown to confer a protective effect [15,16]. In contrast, alcohol consumption and tobacco use are consistently associated with increased colorectal cancer risk, underscoring the critical importance of lifestyle modifications in colorectal cancer prevention [17,18].

The treatment of colorectal cancer is directed by tumor type, stage, and the patient’s overall medical history [19]. Early detection is critical as it enables the implementation of more effective therapeutic strategies, often leading to improved prognoses. For resectable colorectal cancer, surgical excision remains the cornerstone of treatment. In cases of non-resectable colorectal cancer, standard therapeutic modalities include chemotherapy, radiotherapy, and immunotherapy [20]. However, these conventional approaches are associated with significant limitations, including non-specific cytotoxic effects on healthy tissues. Moreover, more than 50% of patients experience disease relapse due to the emergence of acquired multidrug resistance [21]. To address these challenges, recent advancements in colorectal cancer therapeutics have focused on strategies to overcome drug resistance and enhance tumor responsiveness to treatment. These include the development of immune checkpoint inhibitors (ICIs), chimeric antigen receptor (CAR) T cell therapy, T cell receptor (TCR) modifications, cytokine-based therapies, and monoclonal antibodies [19]. In addition, innovative approaches such as probiotics, RNA-based therapies, oncolytic viral therapies, and natural product-based treatments have demonstrated promising potential in preclinical and early clinical studies [22,23,24,25]. Emerging evidence also highlights the role of dietary factors in colorectal cancer prevention and treatment. Specifically, green tea and its bioactive constituents have attracted interest for their potential anticancer effects, underscoring the need for further research into dietary interventions as complementary strategies in colorectal cancer management [26].

Green tea, constituting approximately 20% of global tea production, is one of the most commonly consumed beverages globally. Derived from the *Camellia sinensis* plant, tea is cultivated in over 30 countries, with China, India, Kenya, Sri Lanka, Taiwan, and Japan recognized as leading producers. Unlike white tea, which undergoes minimal processing and is derived from young leaves and buds, or black tea, which involves full fermentation, green tea is produced from more mature leaves and is distinguished by the lack of fermentation during its processing [27,28]. The chemical composition of green tea varies based on environmental conditions such as soil quality, climate, light exposure, geographic factors, microbial interactions, and temperature [29]. Green tea is available in several varieties, each distinguished by unique flavor profiles and antioxidant properties. The method of tea extraction significantly influences its antioxidant potential.

Green tea consumption is associated with diverse biological activities, including antiobesity, antioxidant, and anticancer effects. The antineoplastic properties of green tea are predominantly attributed to its polyphenol content, particularly epigallocatechin-3-gallate (EGCG), the most potent catechin present in green tea [30]. EGCG has been demonstrated to exert chemopreventive effects by targeting multiple stages of carcinogenesis, such as initiation, promotion, and progression. This compound modulates critical cellular signaling pathways that regulate essential processes, including cell proliferation, apoptosis, and angiogenesis [31]. In addition to its role as a chemopreventive agent, EGCG exhibits significant antioxidant activity. It interacts with endogenous antioxidants such as glutathione and generates reactive oxygen species (ROS) to activate pro-oxidative pathways, leading to selective induction of cancer cell death. This dual antioxidant and pro-oxidant functionality underscores the therapeutic potential of EGCG in cancer prevention and treatment [26].

This article aims to provide a comprehensive overview of green tea’s in vitro and in vivo effects, focusing on its potential therapeutic benefits in colorectal cancer. Particular attention was paid to the analysis of clinical trials and meta-analyses related to the use of green tea or green tea catechins in humans.

## 2. Methods

PubMed, Google Scholar, and Web of Science searches were performed to collect the published data using the keywords of green tea, phytochemical, chemical composition, EGCG, pharmacology, tea polyphenols, antioxidants, colon cancer, colorectal cancer, and toxicology. Several websites and related articles were also incorporated. Additionally, some selected articles were manually searched. The inclusion criteria for this review encompassed systematic reviews and experimental studies on green tea, with no restrictions on the publication timeframe.

**Figure 1:**
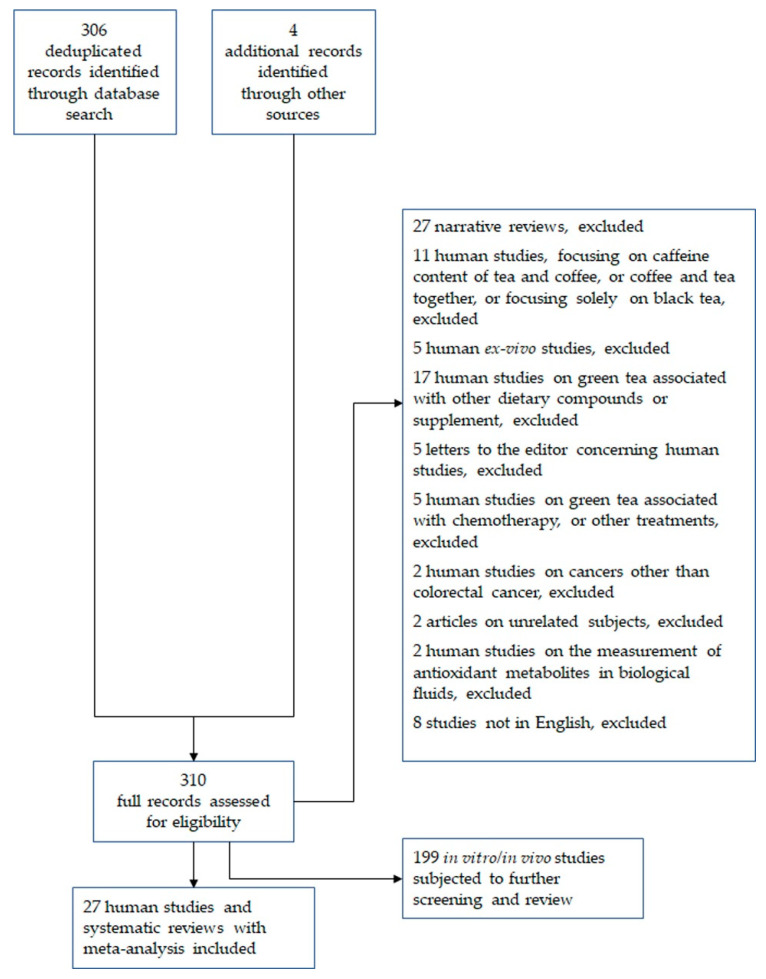
Flowchart of the search and screening process.

An in-depth literature search was also conducted to identify relevant human studies investigating the association between green tea consumption (or green tea extracts) and colorectal cancer risk. Searches were performed across several electronic databases, including Medline, PubMed, and Embase, covering articles in English, published between 1 January 1994 and 30 November 2024. A combination of medical subject headings (MeSH) terms was used as a search strategy to ensure comprehensive retrieval of relevant literature. Key search terms included “cancer*”, “colorectal”, “carcinoma*”, “Green Tea”, “Tea”, and “Catechin*”. A flowchart of the search and screening process is shown in Figure 1.

## 3. Green Tea Constituents

Since the early 20th century, over 500 chemical compounds have been isolated from tea, many of which are associated with beneficial effects on human health [32]. In particular, these bioactive molecules have demonstrated a range of pharmacological properties, including antihypertensive, antihyperlipidemic, antiarteriosclerotic, antidiabetic, anticancer, and antioxidative effects, as well as neuroprotective and blood glucose-lowering activities [33,34,35,36,37].

**Figure 2:**
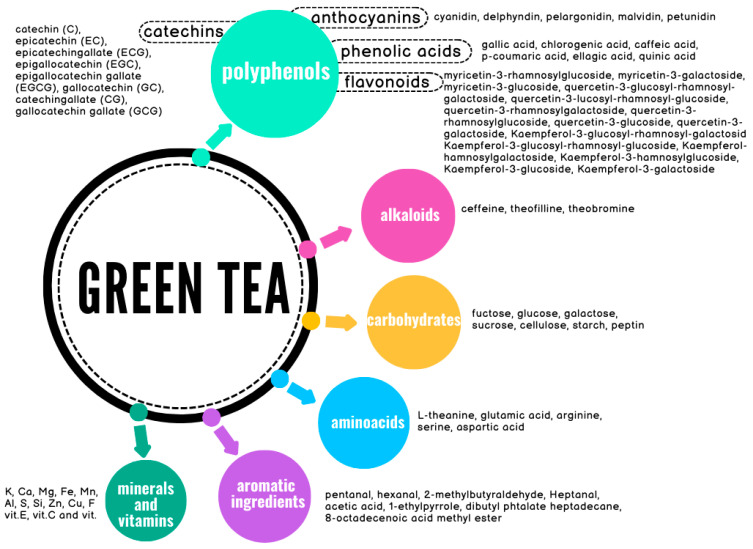
Summary of the main green tea constituents.

The primary constituents of green tea include polyphenols, alkaloids, carbohydrates, amino acids, aromatic compounds, and various mineral elements (Figure 2) [38,39].

Green tea polyphenols, including catechins, flavonoids, anthocyanins, and phenolic acids, constitute the primary bioactive components of green tea leaves and account for 20–30% of the total dry leaf mass [40]. Among these, catechins are the most abundant and are widely regarded as the primary contributors to the biological activities observed in green tea studies [32,41]. Catechins identified in green tea include catechin (C), epicatechin (EC), epigallocatechin (EGC), epicatechin gallate (ECG), gallocatechin (GC), catechin gallate (CG), gallocatechin gallate (GCG), and epigallocatechin gallate (EGCG). Notably, EGCG comprises approximately 60% of the total catechin content, followed by EGC (about 20%), ECG (about 14%), and EC (about 6%) [42,43]. Notably, black tea also contains specific polyphenolic flavonoids exerting antioxidant activities, such as theaflavin-3-gallate.

In addition to catechins, green tea is rich in flavonoids, such as myricetin, quercetin, and behenyl glycosides. Although anthocyanins, a family of water-soluble pigments, are present in low concentrations, they contribute to the bitter taste of tea, significantly influencing its quality [32,44]. The primary alkaloids in green tea are purine alkaloids, with caffeine being the most prevalent, accounting for 2–5% of the total content. Trace amounts of theophylline and theobromine are also present. Together, these three alkaloids constitute the principal components responsible for tea’s invigorating effects [32,45].

Among the primary factors influencing the quality of tea, its amino acid composition plays a pivotal role. Tea typically contains between 1% and 4% amino acids, with a total of 26 distinct amino acids identified to date. These include 20 proteinogenic amino acids and 6 non-proteinogenic amino acids. Prominent amino acids within tea include theanine, glutamic acid, arginine, serine, and aspartic acid. Notably, theanine and γ-aminobutyric acid (GABA) are bioactive compounds in tea that have garnered attention for their neuroprotective properties. Theanine accounts for approximately 50% of the total amino acids present in tea, while GABA is typically found in comparatively lower concentrations.

The aroma of green tea predominantly originates from volatile aromatic compounds, which, although comprising only 0.005% to 0.020% of its chemical composition, exhibit remarkable complexity. Several studies have investigated the volatile constituents of green tea, continually identifying and characterizing novel compounds. In addition to volatiles, water-soluble organic acids significantly contribute to the aroma and flavor of green tea infusions. To date, over 40 organic acids have been identified in tea, including both free organic acids within the infusion and more than 30 associated with aromatic components. Volatile acids such as acetic acid, butyric acid, and hexenoic acid are classified among the aromatic substances responsible for the characteristic scent profile of green tea. Furthermore, green tea contains phenolic acids, including gallic acid, chlorogenic acid, caffeic acid, p-coumaric acid, ellagic acid, quinic acid, tea gallate, and proanthocyanidins. Although these compounds are present in relatively low concentrations, they may contribute to green tea’s sensory attributes and potential health benefits. Research focused on the phenolic acid content and its implications within green tea remains limited, warranting further scientific exploration [32,44].

The slight sweetness of tea infusion is attributed to the presence of small quantities of monosaccharides and disaccharides, including glucose, fructose, galactose, and sucrose. However, the majority of carbohydrates in tea are polysaccharides such as cellulose, starch, and pectin, which are predominantly water-insoluble.

The inorganic constituents of tea, commonly referred to as “ash”, consist primarily of various mineral elements and their oxides. Ash content serves as a critical parameter in the quality evaluation of tea, particularly for export standards. The most abundant mineral elements include phosphorus (P) and potassium (K), followed by calcium (Ca), magnesium (Mg), iron (Fe), manganese (Mn), aluminum (Al), sulfur (S), and silicon (Si). Trace elements such as zinc (Zn), copper (Cu), and fluoride (F) are also present. These mineral elements not only play essential roles in the physiological functions of tea plants but are also recognized for their potential health benefits in humans, prompting significant scientific investigation.

In addition to its complex chemical composition and micronutrient content, green tea contains a variety of vitamins, including vitamin B, vitamin C, and vitamin E. Moreover, it is a source of active enzymes such as glucosidases and lipoxidases, as well as chlorophyll, a natural edible pigment. These components collectively contribute to the nutritional and sensory attributes of green tea and underscore its value as a functional beverage [32,46,47].

## 4. Green Tea Activities

**Figure 3:**
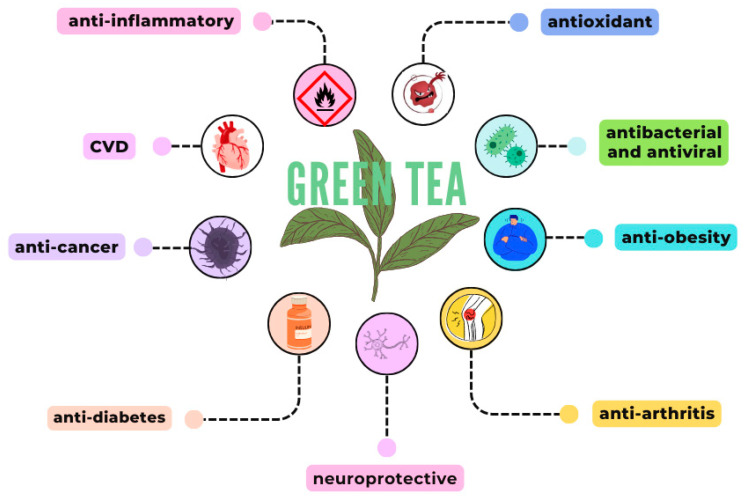
Green tea activities.

The majority of the documented biological effects of green tea, summarized in Figure 3, have been primarily attributed to its catechin content. Nevertheless, additional components of green tea, such as theanine and caffeine, have also been implicated in several physiological activities, including antioxidant effects [48]. As previously mentioned, among the catechins, EC, EGC, ECG, and EGCG are the most abundant and biologically active. These catechins contribute to a wide spectrum of health-promoting effects, including antioxidant, antitumor, anti-inflammatory, antimicrobial, antiviral, antidiabetic, antiobesity, and hypotensive properties [49,50,51]. The synergistic interaction between catechins and other bioactive compounds in green tea underscores its potential as a multifunctional dietary agent with significant therapeutic applications. Further research is warranted to elucidate the specific contributions and mechanisms of these components in mediating green tea’s health benefits.

Green tea’s anti-inflammatory effects are primarily attributed to its capacity to inhibit protein denaturation and enhance the production of anti-inflammatory cytokines. These properties hold therapeutic potential in various diseases characterized by chronic inflammation, including arthritis, cardiovascular diseases, diabetes, obesity, and cancer [51,52]. Moreover, green tea’s combined anti-inflammatory and antioxidant activities contribute to its neuroprotective effects, with evidence suggesting that its bioactive metabolites can cross the blood-brain barrier [53,54]. These properties also underlie green tea’s antibacterial efficacy and its role in the management of cardiovascular diseases (CVD) [55].

Regular consumption of green tea has been demonstrated to offer cardioprotective benefits by preventing atherosclerosis, reducing total cholesterol levels, and improving lipid profiles through a more favorable ratio among low-density lipoprotein (LDL) and high-density lipoprotein (HDL) [51,52]. This multifaceted functionality underscores green tea’s potential as a dietary intervention for both the prevention and management of inflammatory and metabolic disorders.

## 5. Anticancer Effects of Green Tea

**Figure 4:**
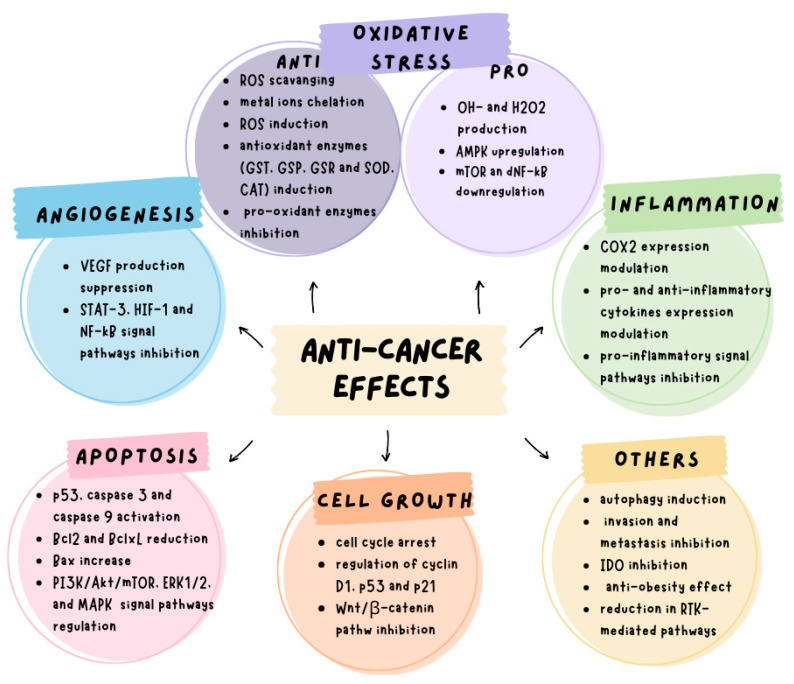
Summary of the anticancer effects of green tea.

Among the various types of tea, green tea has been the most extensively studied for its cancer-preventive and therapeutic properties [41,51,56]. Its anticancer effects are predominantly attributed to catechins, with EGCG exhibiting the highest activity, followed by ECG, EGC, and EC [51]. The biological effects of green tea catechins on cancer cells are mediated through multiple mechanisms, including the induction of apoptosis, cell cycle arrest, inhibition of angiogenesis, and modulation of antioxidant and pro-oxidant activities (Figure 4) [32,35,41]. Neutralization of reactive oxygen species (ROS) is a critical mechanism by which green tea catechins inhibit cancer pathogenesis. These compounds demonstrate potent antioxidant activity by scavenging free radicals, chelating transition metals, and upregulating the expression and activity of antioxidant enzymes such as superoxide dismutase (SOD), catalase (CAT), glutathione-S-transferase (GST), glutathione peroxidase (GSP), and glutathione reductase (GSR) [30,57]. Additionally, green tea catechins help prevent cellular and tissue oxidative damage by inhibiting the activity of pro-oxidant enzymes, including cyclooxygenase (COX) and xanthine oxidase [58].

Interestingly, recent evidence suggests that the anticancer effects of green tea may be more pronounced under conditions of excessive oxidative stress, highlighting the potential context-dependent nature of its therapeutic benefits [32,35,36,41,51]. In this regard, green tea polyphenols exhibit pro-oxidant activity through self-oxidation processes that generate hydroxyl radicals, hydrogen peroxide (H_2_O_2_), and quinonoid intermediates [35,41,59]. This pro-oxidant behavior contributes to their anticancer effects by upregulating *AMP-activated protein kinase* (*AMPK*), which subsequently downregulates *mTOR* and then *nuclear factor of the κ-chain in B-cells* (*NF-κB*) signaling pathways. These pathways are critical for the antineoplastic effects of green tea catechins. Reactive oxygen species (ROS) generated by green tea are integral to the induction of apoptosis, which inhibits cancer cell growth [30]. The pro-apoptotic activity of green tea polyphenols has been linked to their capacity to activate cell death receptors, regulate caspase-8, -3, and -9, and modulate the expression of key apoptosis-regulatory proteins. Specifically, green tea reduces c-Myc, Bcl-2, and Bcl-xL while upregulating Bax, BAD, Apaf-1, Bak, and PUMA protein levels [30,35,60]. Additionally, EGCG has been shown to influence several signaling pathways involved in the regulation of apoptotic cell death, including the *PI3K/AKT/mTOR* (*phosphatidylinositol 3 kinase/protein kinase B/molecular target of rapamycin*), *ERK1/2* (*extracellular signal-regulated kinase 1/2*), and *MAPK* pathways [61].

Autophagy, another critical mechanism in green tea’s anticancer effects is activated by polyphenols, likely through the transcription factor Nrf-2 (nuclear factor erythroid 2-related factor 2). This activation has been shown to inhibit the growth of several cancer cell lines [42,62,63].

Green tea polyphenols also inhibit cancer progression by arresting the cell cycle. This effect is predominantly associated with G0/G1 phase arrest, potentially mediated by the regulation of *cyclin D1*, *cdk4*, and *WAF1/p21* expression [32,64]. However, G_2_/M phase arrest has also been reported [65]. Furthermore, inhibition of the Wnt/β-catenin signaling pathway has been implicated in EGCG-mediated control of cancer cell proliferation [66].

Green tea catechins also repress tumor angiogenesis. This is achieved by inhibiting the activation of *NF-κB*, *STAT3*, *HIF-1*, and the expression of *vascular endothelial growth factor* (*VEGF*) [30,51]. In addition to their antiangiogenic activity, green tea catechins regulate key processes such as epigenetic modification [67], tumor cell migration, and invasion. These effects are facilitated by modulating the expression of *matrix metalloproteinase-9* (*MMP-9*) and its endogenous inhibitor TIMP-1.

Further insights into the molecular mechanisms of catechins, particularly EGCG, reveal their interaction with (67)-laminin receptors, leading to downregulation of *receptor tyrosine kinase* (*RTK*)-mediated signaling pathways [30,51,68]. EGCG also inhibits the expression of *indoleamine 2,3-dioxygenase* (*IDO*), thereby reversing *IDO*-induced immune tolerance and restoring effective antitumor immunity [35]. The chemopreventive properties of green tea extend to its antiobesity effects, which ameliorate metabolic syndrome, a condition linked to increased carcinogenesis risk [69]. Furthermore, emerging evidence highlights EGCG as a potent epigenetic modulator that rectifies abnormal epigenetic events implicated at various stages of carcinogenesis [41].

Green tea’s strong anti-inflammatory effects further contribute to its anticancer activity. These effects involve the suppression of pro-inflammatory cytokine production, inhibition of *cyclooxygenase-2* (*COX-2*) expression, and attenuation of inflammatory signaling pathways such as *STAT3* and *NF-κB* [30,70,71].

## 6. Effects of Green Tea on Colorectal Cancer

As highlighted previously, green tea and its bioactive components have been extensively investigated for their potential antineoplastic effects, particularly in colorectal cancer. While genetic predispositions significantly contribute to the etiology of colorectal cancer, lifestyle factors, particularly dietary habits, are increasingly recognized as modifiable determinants of cancer risk. Green tea, primarily due to its rich composition of polyphenolic compounds, has demonstrated protective effects against the development of multiple cancer types, including esophageal, lung, prostate, stomach, breast, pancreatic, intestinal, and bladder cancers [41,72,73]. Notably, epidemiological evidence has established a correlation between the dietary intake of green tea and a reduced risk of colorectal cancer [74,75,76,77].

**Figure 5:**
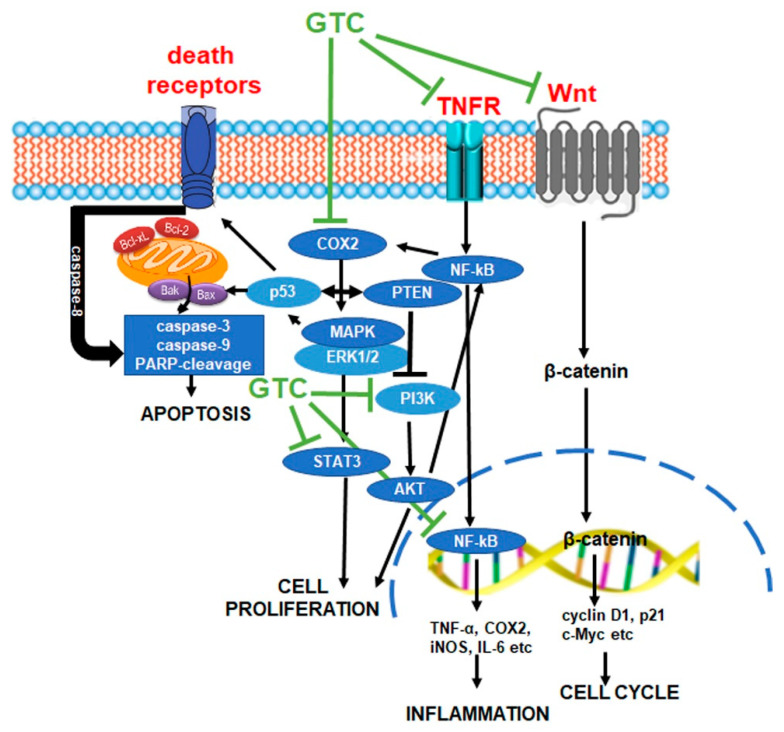
Main mechanisms of action of green tea constituents (GTC) in colorectal cancer.

Over the years, several in vitro and in vivo studies utilizing animal models have investigated the anticancer effects of green tea components, with a primary focus on catechins, in the context of colorectal cancer. A comprehensive summary of these principal studies, including the active principles evaluated and the proposed anticancer mechanisms, is presented in Table 1. Furthermore, a simplified schematic representation of the mechanisms through which green tea exerts its anticancer effects on colorectal cancer is illustrated in Figure 5, providing an integrative overview of its multifaceted action.

### 6.1. In Vitro Studies

Most in vitro studies investigating the anticancer properties against colorectal carcinoma have predominantly focused on catechins, with a particular emphasis on epigallocatechin-3-gallate (EGCG). Notably, two independent studies conducted by Luo et al. demonstrated that EGCG treatment significantly induces apoptosis and suppresses cellular proliferation in SW480, SW620, and LS411N colon cancer cell lines. These effects were mediated through the activation of caspase-3 and poly (ADP-ribose) polymerase (PARP), alongside the downregulation of phosphorylated signal transducer and activator of transcription 3 (p-STAT3). Additionally, EGCG was observed to reduce the expression of the antiapoptotic proteins Bcl-2, Bim, and MCL-1, further elucidating its potential therapeutic mechanisms [78,79]. In the same studies, EGCG has also been reported to exhibit antimigratory effects, potentially mediated by its ability to upregulate *E-cadherin* expression and downregulate Vimentin protein levels [79]. This phenomenon was also observed in a study by Zhang et al., wherein SW480 colon cancer cells co-cultured with neutrophils isolated from colorectal cancer patients demonstrated inhibited migration following EGCG treatment [80]. Beyond the findings reported by Luo et al., other investigators have highlighted the pro-apoptotic activity of EGCG in various colon cancer cell lines [61,81,82,83,84,85]. Several mechanisms have been suggested to explain the pro-apoptotic properties of EGCG. These include the activation of caspase-3, -8, and -9, the upregulation of pro-apoptotic proteins, the downregulation of antiapoptotic proteins, and the suppression of key signaling pathways. Concerning this last issue, EGCG-induced apoptosis and proliferation reduction in DLD1, CaCo-2, HCT116, and HT29 colorectal cancer cell lines, among others, have been associated with the inhibition of different signaling cascades, including the previously mentioned *STAT3* and *AKT/mTOR*, *ERK1/2*, *Wnt/β-catenin*, *MAPK*, and *NF-κB* pathways [61,79,82,84,85,86].

The ability of green tea to inhibit colorectal cancer cell proliferation has also been linked to cell cycle dysregulation. Several studies have demonstrated that EGCG induces cell cycle arrest, primarily at the G0/G1 phase, through the downregulation of *Cyclin D1* expression [81,85,87]. However, G2/M phase arrest has also been observed in some contexts [81,88].

In a recent study, Lin et al. reported that another green tea component, namely, gallic acid (GA), also inhibited proliferation and induced apoptosis in HCT116 and HT29 colon cancer cell lines. These effects were predominantly attributed to an increased ratio of cleaved caspase-3/pro-caspase-3 and cleaved caspase-9/pro-caspase-9, as well as a reduction in phosphorylated epidermal growth factor receptor (p-EGFR), AKT, and STAT3 levels [89].

The antiangiogenic potential of EGCG has been highlighted by Jung et al., who demonstrated that treatment with EGCG reduced *vascular endothelial growth factor* (*VEGF*) expression in HT29 cells [82]. Additionally, recent investigations have revealed that EGCG suppresses cancer stem cell markers and diminishes sphere formation capacity in DLD-1, SW480, and HCT116 cell lines [83,84]. Further, EGCG exerts epigenetic regulatory effects, evidenced by reduced DNA methylation in HCT116, HT29, and SW48 cell lines [87].

### 6.2. In Vivo Studies (Animal Models)

Several studies have shown that green tea polyphenols, mainly EGCG, effectively reduce colon cancer cell growth in animal models. These effects are mediated by mechanisms similar to those observed in vitro [78,81,82,90,91,92]. For example, Jin et al. reported that EGCG treatment induced apoptotic cell death and cell cycle alterations in BALB/c nude mice bearing orthotopically established HT29 tumors [81]. Similarly, Luo et al. observed a significant reduction in tumor volume in mice implanted with SW620 tumors following EGCG administration. This antitumor activity was attributed to the downregulation of phosphorylated STAT3 and its associated downstream signaling pathways [78].

Shimizu et al. demonstrated that drinking water supplemented with EGCG effectively suppressed the growth of SW837 human colorectal cancer xenografts in BALB/c nude mice. This effect was mediated through the inhibition of *hypoxia-inducible factor-1* (*HIF-1*) expression, which subsequently disrupted the *VEGF/VEGFR* signaling axis [91]. Similar findings were reported by Jung et al. in an HT29 xenograft model, where EGCG treatment led to *VEGF* downregulation, correlating with the inhibition of *extracellular signal-regulated kinase 1/2* (*ERK1/2*) [82].

In addition, a recent study by Shojaei-Zarghani et al. highlighted the chemopreventive properties of theanine, a component of green tea. Theanine was shown to inhibit tumorigenesis by suppressing the *AKT/mTOR* and *Janus kinase 2* (*JAK2*)/*STAT3* pathways [92]. Furthermore, the previously mentioned study by Li et al. confirmed the in vivo anticancer efficacy of gallic acid (GA) in a xenograft tumor model. GA treatment inhibited tumor growth, induced apoptosis, and reduced the phosphorylation levels of EGFR, STAT3, and AKT [89].

**Table 1:** Summary of the key findings of in vitro (cell-based) and in vivo (animal-based) studies on the use of green tea or its components, mainly EGCG, in colon cancer and the potential mechanisms involved in their effects.

Type of Model	Treatment	Potential Mechanisms	Effects
**In vitro**			
SW480 and colon cancer cells from patients	0–50 µM EGCG for 48 h	*STAT3/CXCL8* signaling pathway	Significant inhibition of SW480 cells migration and invasion [80]
SW480, SW620 and LS411N cells	10, 20 and 40 μg/mL EGCG	Activation of caspase-3 and PARP, downregulation of *STAT3*, and decrease in Bcl-2 protein levels	Significant suppression of cell proliferation and induction of apoptosis [78]
HT29 cells	EGCG	Inhibition of *ERK1/2* signaling and *VEGF* expression	Significant induction of apoptosis, and angiogenesis inhibition [82]
LoVo, SW480, HCT8, HT29 cells lines	0–35 µg/mL EGCG	Induction of cell cycle alterations and inhibition of *Notch* signaling	Significant inhibition of cell proliferation and apoptosis induction [81]
Caco-2 cells	0–80 µM EGCG	Induction of G2/M cell cycle arrest and activation of laminin receptor-mediated myosin phosphatase	Significant inhibition of cell proliferation [88]
HCT116, HT29, SW480, IEC6 cell lines	0–150 µM EGCG	RXRa activity restoration and *RXRa* promoter methylation reduction	Reversal of gene silencing in colon carcinogenesis modulation [87]
HCT116, HEK293, SW480 cell lines	0–80 µM EGCG	*Wnt/β-catenin* signaling suppression	β-catenin phosphorylation and proteasomal degradation promotion [86]
HCT116, HT29, Caco-2, SW480, and SW837 cells	0–50 µM EGCG or polyphenon E	Induction of G1 cell cycle arrest and reduction of AKT, EGFR, and activation of *HER2*	Significant cell growth inhibition and apoptosis induction [85]
SW480 cells	25 and 50 µM EGCG	*EGFR* downregulation	Significant cell growth inhibition [93]
HT29 cells	0–50 µM EGCG	*AKT*, *ERK1/2*, and *p38 MAPK* signaling pathways modulation	Significant induction of apoptotic cell death [61]
HCT116 spheroids	50 µM EGCG for 1 week	*CD133* and *NANOG* and *ABCC1* and *ABCG2* gene expression decrease	Significant decrease in sphere formation, and apoptosis and cell cycle alterations induction [84]
DLD-1 and SW480 spheroids	0–60 µM EGCG	Colorectal CSC properties and *Wnt/β-catenin* pathway inhibition	Significant reduction of cell proliferation and induction of apoptosis in colorectal CSCs [83]
SW480, SW620 and LS411N cell lines	0–100 μg/mL EGCG	Activation of caspase-3 and PARP, decrease in Bcl-2, MCL-1, and vimentin levels, and increase in E-cadherin levels; downregulation of STAT3 and p-STAT3	Significant inhibition of cell proliferation and migration [79]
HCT116 and HT29 cell lines	30–60 µM GA	Activation of caspase-3 and caspase-9, decrease in STAT3, EGFR, and AKT phosphorylation	Cell proliferation inhibition, apoptosis induction [89]
**In vivo (animal models)**			
SW620 xenograft in BALB/c mice	50 and 100 mg/Kg EGCG i.p. for 4 weeks	STAT3 deregulation	Suppression of tumor volume and weight [78]
azoxymethane-induced premalignant lesions in mice	0.01% and 0.1% EGCG in drinking water for 7 weeks	*β-catenin*, *COX2*, and *cyclin D1* expression decrease	Significant inhibition of premalignant lesions development [90]
xenograft of SW837 cells in BALB/c mice	0.01% and 0.1% EGCG in drinking water for 7 weeks	Suppression of *VEGF/VEGFR* signaling activation	Xenograft tumor growth inhibition [91]
xenograft of HT29 cells in BALB/c mice	5, 10, and 20 mg/Kg/die EGCG intragastrically for 14 days	Inhibition of *Notch* signaling	Significant xenograft tumor volume reduction, and apoptosis induction [81]
xenograft of HT29 cells in BALB/c mice	1.5 mg/day EGCG	VEGF decrease	Significant tumor growth and microvessels density reduction; apoptosis induction [82]
1,2-dimethylhydrazine-induced colon cancer in Wistar rats	400 mg/Kg Theanine for 2 weeks	Ki-67, AKT/mTOR, and JAK2/STAT3 reduction; Smad2 tumor suppressor increase	Precancerous and cancerous lesions, tumor volume reduction [92]
xenograft of HCT116 and HT29 cells in BALB/c mice	80 mg/Kg/day GA	Activation of caspase-3 and caspase-9; decrease in STAT3, EGFR, and AKT phosphorylation	Xenograft volume reduction, apoptosis induction [89]

## 7. Human Studies

This chapter summarizes and synthesizes the findings from studies investigating the relationship between green tea consumption and colorectal cancer risk. In particular, meta-analyses and major studies according to specific geographic regions are reported.

### 7.1. Meta-Analyses

A 2023 systematic review and meta-analysis by Huang et al. assessed the association between tea consumption and colorectal cancer risk across multiple population-based studies. The analysis included data from 15 studies with over 2 million participants [94]. The pooled relative risk (RR) for individuals consuming tea regularly (compared to non-consumers) was 0.76 (95% CI, 0.49–1; *p* = 0.02), indicating a non-significant lowering of the risk of developing CRC. American subgroup analysis confirmed this evidence (RR, 0.32; 95% CI, 0.11–0.91), whereas data from the UK (RR, 1.454; 95% CI, 1.031–2.050) and Italian subgroups (RR, 1.15; 95% CI, 0.087–1.23) showed non-significant risk ratios. Thus, green tea consumption may be associated with a reduction in colorectal cancer risk, although the effect may vary by population.

In a previous meta-analysis by Zhu and colleagues, data from 20 prospective cohort studies involving 2,068,137 participants and 21,437 colorectal cancer cases were analyzed. The overall relative risk for colorectal cancer comparing the highest vs. lowest tea consumption was 0.97 (95% CI, 0.94–1.01; *p* = 0.09), indicating a non-significant association between tea consumption and colorectal cancer risk across all studies [95]. Stratified analysis by geographic region, tea type, age, or smoking showed no significant differences between subgroups. Gender-specific meta-analysis indicated no significant association between tea consumption and colorectal cancer risk in men and a borderline-significant inverse relationship in women (RR with no adjustment for coffee intake, 0.9; 95% CI, 0.82–1.00; *p* < 0.05), suggesting a trend towards protective effect. Overall, conclusions of the study do not associate tea consumption to colorectal cancer risk reduction for both genders combined, though a marginally significant inverse association may exist in women.

A 2017 dose–response meta-analysis from China evaluated the relationship between tea consumption and colorectal cancer risk by pooling 29 studies. The summary odds ratio (OR) suggested a mild but non-significant protective effect of tea on colorectal cancer risk, with moderate heterogeneity observed (OR, 0.93; 95% CI, 0.87–1.00; I^2^ = 43.4%) [76]. Stratified analysis according to study design revealed that green tea had a protective effect in patients with rectal cancer (OR, 0.91; 95% CI, 0.85–0.99). A significant inverse relationship between a 1-cup/day increase in tea consumption and colorectal cancer risk was shown among green tea drinkers (OR = 0.87; 95% CI, 0.76–0.98), and particularly in women (OR, 0.86; 95% CI, 0.78–0.94). In contrast with this evidence, a 2014 dose–response meta-analysis of prospective studies, which evaluated the effect of tea consumption on five major cancers, including breast and colorectal cancers, found no overall inverse association between tea consumption and cancer risk. The study concluded that tea consumption does not appear to have a protective role in preventing five major cancers, including colorectal cancer [96].

A systematic meta-analysis by Wang et al. included six prospective cohort studies, involving 352,275 participants and 1675 cases of colorectal cancer from Eastern Asian countries (Shanghai, Japan, Singapore) [97]. The analysis compared the highest versus lowest levels of green tea consumption and assessed the effect of increasing consumption by one cup per day. The overall relative risk for the highest compared to the lowest green tea consumption was 0.90 (95% CI, 0.72–1.08), suggesting no significant protective effect of green tea against colorectal cancer. Interestingly, considerable heterogeneity, found across the cohort studies for overall risk of colorectal cancer (I^2^ = 59.6%; *p* = 0.011), disappeared after stratification by geographic region, suggesting the presence of ethnic variations in the response of Eastern Asians to tea consumption. Subgroup analysis conducted according to geographic region showed a significant inverse association in the Shanghai population (women and men), where green tea consumption was linked to a 30% reduced risk of colorectal (RR, 0.70; 95% CI, 0.55–0.85) and colon cancers (RR, 0.69; 95% CI, 0.48–0.98). Similar to the meta-analyses summarized above, the protective effect against colorectal cancer was more pronounced in women (one study, RR, 0.63; 95% CI, 0.45–0.88). In contrast, a 36% higher risk of colorectal cancer was found in Singaporean men (one study, RR, 1.36; 95% CI, 1.06–1.74) [97].

By pooling 13 cohort studies, a 2010 USA meta-analysis examined the association between the risk of colorectal cancer and the consumption of coffee, tea, and sugar-sweetened carbonated soft drinks. The analysis included 731,441 participants followed for 6 to 20 years, with 5604 colon cancer cases assessed. No significant association was found between colon cancer risk and coffee consumption or carbonated drinks. Tea consumption showed a modest, significant association with colon cancer risk, with a pooled RR of 1.28 (95% CI, 1.02–1.61; *p* = 0.01) for those drinking more than four 8 oz cups daily. These associations did not differ according to the tumor site, body mass index, physical activity, alcohol use, smoking status, or sex. The study concluded that a modest positive association with colorectal cancer in the case of higher amounts of tea consumed was observed, although data on the type of tea in most studies were not available [98].

A 2012 meta-analysis of case–control studies included 13 studies involving 12,636 cases and 38,419 controls, extracted from studies performed in Asia, North and South America, and Europe [99]. Pooled analysis revealed a non-significant reduction in colorectal cancer risk associated with high green tea intake (OR, 0.87; 95% CI, 0.48–1.58; *p* = 0.65). Analysis was characterized by very high heterogeneity (I^2^ = 90.9; *p* < 0.001). Stratification by continent did not result in significant odds ratios. Thus, the data presented in this meta-analysis were insufficient to definitively establish green tea as a protective factor against human colorectal cancer.

In conclusion, while the overall evidence from meta-analyses focusing on tea consumption and CRC risk remains uncertain, a consistent trend suggests a protective effect, particularly in women. Further research, particularly focusing on gender differences and geographic variations, may confirm and refine these findings.

Table 2 summarizes the meta-analysis evidence on the relationship between green tea consumption and colorectal cancer, obtained in the studies considered.

### 7.2. Major Studies According to Specific Geographic Regions

#### 7.2.1. Asian Studies

##### Japan

A case–control analysis performed in the frame of the Fukuoka colorectal cancer study investigated the association between dietary intake of polyphenols and colorectal cancer risk in 816 colorectal cancer cases and 815 community-based controls. Polyphenol intake was assessed from 97 food items using the Phenol-Explorer database, specifically focusing on tea and coffee polyphenols [100]. There was no correlation found between tea polyphenols and colorectal, colon, or rectal cancer (OR adjusted for age, sex, residential area, familial history, smoking, alcohol, BMI, profession, and physical activity, 1.37; 95% CI, 0.99–1.90; *p* = 0.09).

In a population-based prospective cohort study, the association between green tea consumption and colorectal cancer risk was evaluated in 13,957 men and 16,374 women from Japan over a 12-year follow-up period. The study found no overall significant association between green tea consumption and colorectal cancer risk in both men and women. However, there was a weak inverse correlation between men with increased green tea consumption and their colon cancer risk. Compared to men consuming no or less than one cup of green tea per day, the relative risks for colon cancer were 1.32, 0.76, and 0.78 for those consuming 1, 2–3, and 4 or more cups per day, respectively (*p* = 0.045 for a linear trend). While this trend suggests a potential protective effect of higher green tea consumption on male colon cancer risk, no significant association was found [101].

A pilot study by Shimizu et al. (2008) investigated the potential of green tea extracts in preventing metachronous colorectal adenomas. In this trial, participants who had previously undergone polypectomy for colorectal adenomas were randomly assigned to receive either green tea extract or a placebo for 12 months. At end-point colonoscopy, at least one colorectal adenoma was diagnosed in 31% (20 of 65) of the patients in the control group. Supplementation with 1.5 g GTE appeared to prevent metachronous adenomas to 15% (9 of 60 patients; relative risk, 0.49; 95% confidence interval, 0.24–0.99; *p* < 0.05). These findings suggest that green tea extracts may have a protective effect against the recurrence of colorectal adenomas, though the study called for larger trials to confirm the efficacy and explore the mechanisms behind this effect [102].

In a study conducted in 2005, Suzuki and coworkers pooled two prospective cohort studies conducted in northern Japan, including a total of 65,915 subjects. The analysis showed no significant association between green tea consumption and colorectal cancer risk [103]. In particular, the hazard ratio (HR) for colon cancer for those drinking five or more cups of green tea per day, compared to fewer than one cup, was 0.97 (95% CI, 0.70–1.35). Similarly, for rectal cancer, the HR was 0.85 (95% CI, 0.58–1.23).

Thus, studies focusing on the Japanese population found contradictory evidence concerning the relationship between green tea consumption and the risk of colorectal cancer.

##### Korea

A case–control study conducted in Korea examined the association between green tea intake and colorectal cancer risk, considering the influence of lifestyle factors. The study included 922 cases and 1820 controls. The results showed that participants in the highest tertile of green tea intake showed significantly lower odds of colorectal cancer compared to those in the lowest tertile (OR, 0.48; 95% CI, 0.39–0.59; *p* < 0.001) [104]. Regardless of lifestyle changes, a high green tea intake was consistently associated with a lower risk of colorectal cancer when green tea consumption and lifestyle factors interactions were considered.

The ability of green tea extract (GTE) supplements to prevent metachronous colorectal adenomas and cancer in 176 participants who had undergone complete removal of colorectal adenomas by endoscopic polypectomy was evaluated in a randomized controlled trial also conducted in Korea. Participants were randomly assigned to receive either 0.9 g of GTE per day for 12 months or no GTE supplementation. The incidence of metachronous adenomas was significantly lower in the GTE group (23.6%) compared to the control group (42.3%), with a relative risk (RR) of 0.56 (95% CI, 0.34–0.92; *p* = 0.018). Additionally, the GTE group experienced fewer relapsed adenomas (0.3 ± 0.6) compared to the control group (0.7 ± 1.1), with a statistically significant difference (*p* = 0.010) [105]. Similarly to the results of the Japanese trial reported by Shimizu and coworkers [102], GTE supplementation appears to have a favorable chemopreventive effect on metachronous colorectal adenomas in Korean patients.

##### Singapore

The Singapore study, which was the main outlier in the Wang et al. (2012) meta-analysis described in the previous chapter, was a prospective cohort study examining the relationship between green or black tea consumption and colorectal cancer risk in 60,000 men and women. After an average follow-up of 8.9 years, 845 colorectal cancer cases were identified [74]. Although green tea drinkers had a statistically non-significant increased colorectal cancer risk compared to non-drinkers in the overall population, a significant risk increase was calculated in men (RR, 1.31; 95% CI, 1.08–1.58), while women showed no significant association. Among men, green tea was particularly strongly associated to colorectal cancer for advanced stages of the disease, with an RR of 1.53 (95% CI, 1.19–1.97) and showed a dose-dependent relationship (*p* for trend = 0.0002). Black tea intake showed no significant association with colorectal cancer risk for either gender (RR, 0.92; 95% CI, 0.79–1.07). Thus, in contrast with other studies performed on various Asian populations, these findings suggest that green tea consumption may be linked to a slightly higher risk of colorectal cancer and that such an effect may be related to the genetics of the specific population tested.

##### Taiwan

In a cross-sectional study by Chen et al., the association between cumulative tea consumption and colorectal adenomas was examined in a Taiwanese population of 1688 adults who underwent colonoscopy screening [106]. The variable “cup of tea” used in this study was obtained by multiplying the daily cups with the years of tea consumption. A negative association was found between tea consumption and the likelihood of high-risk adenomas in the group in which the largest amount of tea was consumed (≥42 cup-years) compared to non-consumers (OR, 0.57; 95% CI, 0.36–0.89; *p* = 0.02). Similar results were obtained for low-risk adenomas (OR, 0.66; 95% CI, 0.48–0.90; *p* = 0.01), even for consumers of lower amounts of tea (e.g., 12–41.9 cup-years; OR, 0.64; 95% CI, 0.46–0.90; *p* = 0.01). These results suggest that cumulative tea consumption may be associated with a significantly reduced risk of colorectal adenomas in the Taiwanese population and are in agreement with the results of the GTE Korean study by Shin et al. [105].

##### China

Two key Shanghai-based studies included in the 2012 meta-analysis by Wang et al., cited in the previous chapter, reported a protective effect of green tea against colorectal cancer. These studies were the main contributors to the significant protective effect observed in the meta-analysis, highlighting green tea’s potential in reducing colorectal cancer risk, particularly in specific subgroups.

The first study, conducted by Ji et al. (1997), examined the association between green tea intake and the risk of colon, rectal, and pancreatic cancers [77]. This study included 931 colon, 884 rectal, and 451 pancreatic cancer cases diagnosed between 1990 and 1993, with 1552 age- and gender-matched controls. Multivariate analyses, adjusted for age, income, education, and smoking, showed an inverse relationship between green tea consumption and cancer risk, particularly for rectal cancer. Among men, those in the 200–299 g/month consumption range had an odds ratio of 0.66 (95% CI, 0.43–0.99) for rectal cancer. No significant associations were found for colon cancer. A more pronounced trend was observed in women, who showed significant protective activity against rectal cancer for both the lower (1–200 g/month, OR, 0.51; 95% CI, 0.33–0.79) and the highest consumption categories (≥200 g/month, OR, 0.57, 95% CI, 0.34–0.97). Non-significant results were found for colon cancer. These findings suggest that green tea consumption may have a preventive activity against the risk of rectal cancer, especially in Chinese women.

Focusing on a cohort of 60,567 Chinese men, the second Shanghai study found that regular green tea consumption (namely, drinking green tea at least three times per week for more than six months) was associated with a reduced risk of colorectal cancer (HR, 0.77; 95%CI, 0.59–1.01), which was more pronounced and statistically significant among non-smokers (multivariable-adjusted HR, 0.54; 95% CI, 0.34–0.86). Interestingly, the more green tea that was consumed, the more the risk decreased. Specifically, a 12% reduction in risk was associated with each 2 g increment in daily intake of dry green tea leaves (HR, 0.88; 95% CI, 0.78–0.99). No significant association was found between green tea consumption and colorectal cancer risk among smokers (HR, 0.94; 95% CI, 0.66–1.34) [75]. This study suggested that regular green tea consumption may reduce the incidence of colorectal cancer, particularly among non-smokers.

A prospective cohort study, which included 69,710 Chinese women aged 40 to 70 years, evaluated the association between green tea consumption and colorectal cancer risk. Baseline in-person interviews were used to assess tea consumption and were reassessed 2–3 years later. Over 6 years of follow-up, 256 new cases of colorectal cancer were identified. Women who reported moderate consumption of green tea (1–4 g/day) showed an age-adjusted relative risk of colorectal cancer of 0.64 (95% CI, 0.44–0.94) compared to non-regular tea drinkers. Women drinking higher amounts of tea (>/=5 g/day) had a lower relative risk of colorectal cancer (0.51; 95% CI, 0.29–0.89; *p*/trend = 0.002). Both colon and rectal cancers showed this inverse association [107].

Negative results were reported by Li et al., who conducted a large prospective cohort study within the China Kadoorie Biobank to assess the relationship between tea consumption and cancer risk among 455,981 Chinese adults aged 30–79 years. Data, adjusted for confounders such as alcohol consumption and smoking, showed that regular green tea drinkers did not show a decreased hazard of developing cancer overall (HR, 1.03; 95% CI, 0.93–1.13). This negative finding included all kinds of assessed cancers, including colorectal cancer (daily consumers of tea leaves >4.0 g/day vs. less-than-weekly consumers: HR, 1.08; CI, 0.81–1.45) [108].

Studies conducted in China explored the relationship between genetic susceptibility to CRC and environmental factors, such as diet and, in particular, tea consumption.

An interesting population-based case–control study, including 421 colorectal cancer patients and 845 controls, was conducted in China to assess the association between XPC polymorphisms (Lys939Gln, Ala499Val, and PAT) and colorectal cancer risk [109]. The XPC gene plays a crucial role in repairing bulky, helix-distorting DNA lesions, and several polymorphisms in this gene may influence susceptibility to colorectal cancer [110]. Importantly, the study investigated the impact of green tea consumption on the odds of having colorectal cancer in carriers of specific XPC polymorphisms. For the Lys939Gln polymorphism, the CC genotype showed significantly higher odds for colorectal cancer (OR, 1.5; 95% CI, 1.0–2.2) compared to the AA genotype. Interestingly, those who never drank tea had higher odds ratios (ORs) for the associations between colorectal cancer risk and the AC + CC genotype (OR, 3.0; 95% CI, 1.9–4.7), as well as the AA genotype (OR, 2.4; 95% CI, 1.5–3.9), than tea drinkers with the AA genotype. In addition, the PAT +/+ genotype was also associated with a significantly greater risk of colorectal cancer (OR, 1.5; 95% CI, 1.0–2.3) compared to the PAT −/− genotype. Once more, comparing tea drinkers with the PAT −/− and −/+ genotypes, the genotype impact was more noticeable among never tea drinkers with the PAT +/+ genotype (OR, 3.3; 95% CI, 1.8–6.1), as well as the PAT −/− and −/+ genotypes (OR, 2.5; 95% CI, 1.7–3.6). While no significant association was found between the Ala499Val polymorphism and overall colorectal cancer, the authors observed significantly increased odds among never-tea drinkers carrying either the CC genotype (OR, 2.8; 95% CI, 1.8–4.4) or the CT + TT genotype (OR, 2.2; 95% CI, 1.4–3.3) as compared with tea drinkers carrying the CT + TT genotype [110].

Yu et al. (2012) investigated the influence of gene mutations in the cytosolic phospholipase A2 PLA2G4A gene on the protective effect of tea consumption against colorectal cancer. From a cohort of 1200 individuals, it was found that in carriers of the PLA2G4A rs6666834 polymorphism, tea consumption exhibited a significant protective effect. Compared with non-tea drinkers with the TT/CT genotype of rs6666834, tea drinkers with TT/CT or CC showed significantly lower odds of colorectal cancer (OR, 0.6; 95% CI, 0.36–1.00 for TT/CT [*p* = 0.047]; 0.38, 0.19-0.74 for CC, [*p* = 0.0049]). This suggests that the analyzed genetic variants may influence the cancer preventive properties of tea, underscoring the complex interplay between diet, genetic factors, and cancer susceptibility [111].

These results suggest that genetic variations may predispose individuals to colorectal cancer, but green tea consumption appears to modulate this risk, providing a protective effect for carriers of certain genotypes. In addition, the interplay between genetics and diet could inform personalized prevention strategies for colorectal cancer.

Except for the Singapore study, which showed the opposite trend, most trials conducted in Asia consistently support the conclusion that green tea consumption may be associated with a reduced risk of colorectal cancer. These studies, particularly those from regions like Shanghai, suggest that green tea may confer a protective effect against colorectal cancer, highlighting a potential link between its regular consumption and lower cancer risk. In addition, studies from Korea and Taiwan have demonstrated a protective effect of green tea or its extracts against colorectal adenomas.

#### 7.2.2. European, American, and Australian Studies

##### Europe

Data from the European Prospective Investigation into Cancer and Nutrition (EPIC) cohort, a comprehensive epidemiological study exploring various dietary and lifestyle factors related to cancer risk, were used in two different studies. In one of these, the association between the dietary intake of total and individual polyphenol classes and colorectal cancer (CRC) risk was evaluated in 476,160 European men and women. Polyphenol intake was assessed using country-specific dietary questionnaires. Over a follow-up period of 14 years, 5991 colorectal cancer cases were recorded [112]. The analysis found that a doubling of total dietary polyphenol intake was not associated with colorectal cancer risk in either women (hazard ratio [HR] log2, 1.06; 95% CI, 0.99–1.14) or men (HR log2, 0.97; 95% CI, 0.90–1.05). In a second study by Dik and coworkers, the association between coffee and tea consumption and colorectal cancer risk was investigated using data from 477,071 participants of the EPIC cohort. Habitual coffee and tea consumption was assessed using dietary questionnaires [113]. Over a median follow-up of 11.6 years, 4234 participants developed CRC. Concerning tea consumption, no significant association was found between total consumption and CRC risk (HR, 0.97; 95% CI, 0.86–1.09). Risk estimates for tea consumption and colorectal cancer risk were not modified by sex, diabetes, BMI, and intake of red or processed meat. Furthermore, when considering potential effect modifications by genotypes related to caffeine metabolism (CYP1A2 and NAT2), no significant differences in colorectal cancer risk were observed between high tea consumers with slow enzyme activity and non-/low consumers with fast enzyme activity. These findings suggest that caffeine metabolism does not significantly influence the relationship between coffee and tea consumption and colorectal cancer risk. Overall, both studies from the EPIC cohort found no significant association between polyphenol or tea consumption and colorectal cancer risk, suggesting that these dietary factors may not play a major role in influencing colorectal cancer risk in the European population investigated.

Non-significant results also emerged from a randomized, double-blind trial performed in Germany. The effectiveness of a green tea extract (GTE), standardized to 150 mg of epigallocatechin gallate (EGCG) twice daily, was assessed for preventing colorectal adenomas in 1001 participants with a history of colon adenomas. After a 4-week safety run-in period, participants were randomized to receive either GTE or a placebo for 3 years. There were no significant variations in the adverse events between the two groups, indicating that GTE has a good safety profile. Furthermore, there was no significant reduction of adenoma incidence with GTE in the modified intention-to-treat population. Specifically, the adenoma rate in the placebo group was 55.7%, and in the GTE group, it was 51.1% (adjusted relative risk, 0.905, *p* = 0.16). Similarly, in the per-protocol population, the adenoma rate difference was also not significant (adjusted relative risk 0.883; *p* = 0.117). The study concluded that GTE did not significantly reduce the incidence of colorectal adenomas compared to placebo [114].

Over a median follow-up of 8 years, 111 cases of colon cancer and 83 cases of rectal cancer were diagnosed in Finland. Using proportional hazard regression models, the authors examined the association between coffee and tea consumption and cancer incidence, adjusting for potential confounders. Coffee consumption was not significantly associated with either colon or rectal cancer. However, a positive association was observed between tea consumption and colon cancer risk. Compared to non-tea drinkers, those who consumed more than 1 cup/day had an RR of 2.09 (95% CI, 1.34–3.26). On the other hand, rectal cancer incidence was not significantly improved by tea consumption. Thus, the hypothesis that coffee or tea can protect against colorectal cancer in Finnish men was not supported by this study [115].

In a large Swedish cohort study, Terry and coworkers examined 61,463 women over an average 9.6 years of follow-up and identified 460 incident cases of colorectal cancer. No overall associations were found between tea consumption and colorectal cancer in age- or multivariate-adjusted models. For the highest tea consumption category (two or more cups/day), the multivariate-adjusted relative risk compared to non-consumers was 0.97 (95% CI, 0.63–1.48). No significant association was found for proximal, distal, or combined colon cancer. However, a non-significant positive association with rectal cancer was observed, which became statistically significant in women aged over 65 years (RR, 2.26; 95% CI, 1.03–4.96) [116]. In conclusion, cohort studies conducted in Scandinavian populations yielded mixed results regarding the relationship between tea consumption and colorectal cancer risk. The Finnish study found a positive association between tea consumption and colon cancer risk, while the Swedish study reported no significant overall association, although a marginally significant positive association with rectal cancer risk was observed in women over 65. These findings contrast with data from studies in China, which consistently appear to show a protective effect of tea consumption against colorectal cancer. This suggests that the impact of tea on colorectal cancer risk may vary by population, age, and other factors.

##### USA

In a study by Shi et al. (2023), the association between flavonoid intake and survival after colorectal cancer diagnosis was assessed in two large U.S. cohorts: the Nurses’ Health Study and the Health Professionals Follow-Up Study. The analysis included 3695 CRC survivors and evaluated the impact of flavonoid consumption on overall survival and CRC-specific survival. The results indicated that higher flavonoid intake was associated significant improvements in both overall survival and colorectal cancer-specific survival. Specifically, for every standard deviation increase in total flavonoid intake, the hazard ratio for overall mortality was 0.89 (95% CI, 0.83–0.95), and for colorectal cancer-specific mortality, the HR was 0.84 (95% CI, 0.71–0.98). Flavonoid subclasses, mainly the flavan-3-ol tea component, are particularly associated with improved survival outcomes. The multivariable HRs for CRC mortality per 1 daily cup of tea was 0.86 (0.75–0.99; *p* = 0.03) [117].

A US-based study aimed at examining the relationship between tea consumption and colon cancer risk using data from the NHANES I Epidemiologic Follow-up Study (NHEFS). The researchers applied Cox proportional hazard models to test whether frequent tea consumption might have a protective effect against colon cancer. The study included 7656 tea users and 4514 non-tea users. After adjusting for potential confounders, the relative risks of colon cancer for tea drinkers were significantly lower compared to non-tea drinkers either for those drinking up to 1.5 cups per day (RR, 0.57; 95% CI, 0.42–0.78) or for subjects consuming more than 1.5 cups per day (RR, 0.59; 95% CI, 0.35–1.00). Notably, the protective effect was more pronounced in men, with an RR of 0.41 (95% CI, 0.25–0.66) for men consuming up to 1.5 cups per day and an RR of 0.30 (95% CI, 0.09–0.98) for those consuming more than 1.5 cups per day. In conclusion, this study suggests an inverse relationship between habitual tea consumption and colon cancer risk, particularly among men [118].

A smaller population-based case–control study in Midwestern US included 685 colon cancer cases and 655 rectal cancer cases, aged 40–85 years, identified through the Iowa SEER Cancer Registry. Controls (n = 2434) were frequency-matched by sex and age. Total tea consumption was categorized as none, low (≤3 cups/day), medium (3.1–5 cups/day), and high (>5 cups/day). The study found no association between total tea consumption and either colon or rectal cancer after adjusting for age, sex, education, physical activity, smoking history, and intake of coffee, fiber, and fruits and vegetables [119].

In conclusion, studies conducted in the USA suggest varying associations between tea consumption and colorectal cancer risk. While a study in the Nurses’ Health Study and the Health Professionals Follow-Up Study found that higher flavonoid intake, particularly from tea, was associated with improved survival outcomes after colorectal cancer diagnosis, and the NHANES study showed a marked reduction in colorectal cancer risk, other studies, such as the one based on the Iowa SEER Cancer Registry, did not find a significant protective effect of tea consumption on colorectal cancer risk. These findings highlight the complex and context-dependent nature of the relationship between tea intake and colorectal cancer outcomes in U.S. populations.

##### Australia

A case–control study conducted in Western Australia (2005–2007) analyzed the associations between tea, coffee, and milk consumption and colorectal cancer risk in 854 incident cases and 948 controls. No significant positive or inverse associations were found between black tea, green tea, decaffeinated coffee, or milk and colorectal cancer risk [120]. However, the highest rate of green tea consumption in this study was rather undefined (one or more cups a week), and subgroup analysis in this intake cohort was not performed.

## 8. Discussion

Green tea, traditionally consumed for centuries as a tonic beverage, has only recently become the focus of extensive scientific research aimed at elucidating its potential health benefits, particularly in disease prevention, including cancer. According to epidemiological studies, green tea consumption may lower the risk of developing several malignancies, including colorectal cancer [101,104,120]. Current evidence highlights that green tea catechins, particularly epigallocatechin-3-gallate (EGCG), possess both therapeutic and preventive potential. These compounds inhibit cancer initiation, development, and progression by modulating essential cellular processes such as proliferation, differentiation, apoptosis, angiogenesis, and metastasis [35,41,51].

Studies utilizing tumor cell lines and animal models have provided significant insights into the molecular mechanisms underlying the anticancer properties of green tea catechins. Extensive research has demonstrated that these catechins regulate critical biological pathways involved in colorectal carcinogenesis. Notably, EGCG effectively induces apoptotic cell death and enforces cell cycle arrest in cancer cells while exerting minimal effects on normal cells, emphasizing its selective activity [32,35,41,56]. Furthermore, multiple animal studies have corroborated these findings, revealing that green tea treatment markedly reduces the incidence and growth of colorectal tumors, thereby supporting its potential role as a chemopreventive and therapeutic agent [81,82,85,90].

Evidence gathered from individual studies and meta-analyses consistently indicates that green tea consumption may exert a protective effect against colorectal cancer in specific populations, such as China (Shanghai), Taiwan, and Korea, whereas it appears to be ineffective, or even detrimental (e.g., Singapore), in a number of other ethnic groups. Moreover, the magnitude of this effect appears to vary substantially across studies. This variability is likely attributable to differences in study designs (e.g., case–control versus cohort studies), methods of exposure assessment (e.g., self-reported green tea intake), and the heterogeneity of the study populations. These factors underscore the inherent complexity of investigating dietary interventions in cancer prevention. Geographically, regular green tea consumption has been most strongly associated with a reduced risk of colorectal cancer in East Asian populations, where the intake is typically higher. Moreover, research into gene–environment interactions, such as the influence of genetic polymorphisms related to DNA repair mechanisms, suggests that certain subpopulations may derive enhanced benefits from consistent green tea consumption. However, similar protective associations have not been consistently observed in European populations, highlighting potential regional differences in dietary patterns, genetic predisposition, or environmental factors.

Despite experimental evidence supporting the association between green tea catechin intake and a reduced risk of colorectal cancer, their clinical applications remain limited, primarily due to poor bioavailability. The chemopreventive and anticancer effects of EGCG are strongly influenced by its absorption efficiency and interactions with target tissues. Thus, EGCG’s limited absorption and instability within the gastrointestinal tract significantly impede its ability to effectively reach therapeutic targets, leading to discrepancies between its demonstrated in vitro and in vivo biological effects. Catechins, including EGCG, are rapidly metabolized under physiological conditions, often resulting in the formation of degradation products or pro-oxidant molecules, irrespective of the administration method [41,121,122]. While administering higher doses of catechins might compensate for these bioavailability challenges, excessive intake is associated with dose-dependent toxicological effects. Consequently, achieving an effective therapeutic balance remains a challenge for clinical application [123]. Significant attempts are being made to overcome these limitations and enhance EGCG’s bioavailability and improve its cellular uptake. Among these strategies, encapsulation of EGCG within hydrophobic nanocarriers has shown considerable promise, particularly in colorectal cancer models. Such nanocarrier systems provide increased stability and targeted delivery of EGCG, thereby overcoming traditional barriers to its clinical use [124,125,126,127].

Although not specifically discussed in this review, studies investigating the co-administration of EGCG with other nutraceuticals, such as curcumin, or conventional chemotherapeutic agents, including irinotecan, have yielded promising results [128,129]. These findings suggest that EGCG may serve as a valuable adjuvant in traditional chemotherapy, potentially enhancing the efficacy of chemotherapeutic agents while mitigating their associated side effects. This dual action could improve therapeutic outcomes and contribute to an overall enhancement in the quality of life for patients undergoing cancer treatment.

## 9. Conclusions

Despite the promising findings obtained both in vitro and in vivo, the evidence regarding the use of green tea components, mainly green tea catechins, in colon cancer prevention and treatment remains inconclusive. Some studies have reported no significant associations or, in certain cases, positive associations between green tea consumption and colorectal cancer risk. To establish its efficacy as a chemopreventive agent, rigorous randomized controlled trials and long-term cohort studies are essential. Particular emphasis should be placed on assessing potential sex-specific effects, especially on women, as these may differ due to hormonal and metabolic factors.

Additionally, future research should aim to determine the optimal dosages and durations of green tea catechin intake required for maximum protective benefits while minimizing risks. Investigations into the interactions between green tea and other dietary or lifestyle factors that influence cancer risk would further clarify its role in colorectal cancer prevention and treatment. It would be of significant interest to further investigate the molecular mechanisms underlying the synergistic interactions between EGCG and conventional chemotherapeutic/chemopreventive agents or nutraceutical compounds. Expanding the scope of research to include additional conventional pharmacological agents could provide valuable insights into optimizing their clinical applications. The ability of EGCG to enhance therapeutic efficacy may enable the use of lower drug dosages, thereby minimizing associated adverse effects and improving the overall therapeutic index.

## Figures and Tables

**Table 2:** Meta-analysis evidence on the relationship between green tea consumption and colorectal cancer (RR, risk ratio; OR, odds ratio; 95% CI, 95% confidence interval).

Authors, Year	Number of Pooled Studies	Comparisons	Major Findings	Reference
Huang et al., 2023	15	Regular tea consumption vs. non-consumers	Risk of CRC not significantly decreased in the general population; significant 68% decrease in American subgroup	[94]
Zhu et al., 2020	20	Highest vs. lowest tea consumption	Risk of CRC not significantly decreased in the general population; significant, 10% decrease in women	[95]
Chen et al., 2017	29	Increase in tea consumption (one cup increase)	Odds of CRC not significantly decreased in the general population; significant 9% decreased odds found against rectal cancer; significant, 14% decrease in CRC odds in women stratum	[83]
Yu et al., 2014	15	Risk estimate per 3 cups increase	No significant decrease in CRC risk	[96]
Wang et al., 2012	6	Highest vs. lowest tea consumption	Risk of CRC not significantly decreased in the general population; significant, 30% decrease in risk in Shanghai subgroup; 36% increased CRC risk in Singapore subgroup	[97]
Wang et al., 2012	13		No significant decrease in CRC risk	[99]
Zhang et al., 2010	13	Consumption increment of more than 4 cups/day of tea (type of tea not disclosed)	Significant increase in CRC risk (28%)	[98]

## References

[1] Hossain M.S., Karuniawati H., Jairoun A.A., Urbi Z., Ooi D.J., John A., Lim Y.C., Kaderi Kibria K.M., Mohiuddin A.K.M., Ming L.C. (2022). Colorectal Cancer: A Review of Carcinogenesis, Global Epidemiology, Current Challenges, Risk Factors, Preventive and Treatment Strategies. Cancers.

[2] Saraiva M.R., Rosa I., Claro I. (2023). Early-Onset Colorectal Cancer: A Review of Current Knowledge. World J. Gastroenterol..

[3] Arnold M., Sierra M.S., Laversanne M., Soerjomataram I., Jemal A., Bray F. (2017). Global Patterns and Trends in Colorectal Cancer Incidence and Mortality. Gut.

[4] Sawicki T., Ruszkowska M., Danielewicz A., Niedźwiedzka E., Arłukowicz T., Przybyłowicz K.E. (2021). A Review of Colorectal Cancer in Terms of Epidemiology, Risk Factors, Development, Symptoms and Diagnosis. Cancers.

[5] Mármol I., Sánchez-de-Diego C., Dieste A.P., Cerrada E., Yoldi M.J.R. (2017). Colorectal Carcinoma: A General Overview and Future Perspectives in Colorectal Cancer. Int. J. Mol. Sci..

[6] Fearon E.R., Vogelstein B. (1990). A Genetic Model for Colorectal Tumorigenesis. Cell.

[7] Wells K., Wise P.E. (2017). Hereditary Colorectal Cancer Syndromes. Surg. Clin. N. Am..

[8] Umar A., Boland C.R., Terdiman J.P., Syngal S., de la Chapelle A., Rüschoff J., Fishel R., Lindor N.M., Burgart L.J., Hamelin R. (2004). Revised Bethesda Guidelines for Hereditary Nonpolyposis Colorectal Cancer (Lynch Syndrome) and Microsatellite Instability. J. Natl. Cancer Inst..

[9] Willett C.G., Chang D.T., Czito B.G., Meyer J., Wo J., Cancer Genome Atlas Network (2012). Comprehensive Molecular Characterization of Human Colon and Rectal Cancer. Nature.

[10] Karahalios A., English D.R., Simpson J.A. (2015). Weight Change and Risk of Colorectal Cancer: A Systematic Review and Meta-Analysis. Am. J. Epidemiol..

[11] Van Kruijsdijk R.C.M., Van Der Wall E., Visseren F.L.J. (2009). Obesity and Cancer: The Role of Dysfunctional Adipose Tissue. Cancer Epidemiol. Biomark. Prev..

[12] Vieira A.R., Abar L., Chan D.S.M., Vingeliene S., Polemiti E., Stevens C., Greenwood D., Norat T. (2017). Foods and Beverages and Colorectal Cancer Risk: A Systematic Review and Meta-Analysis of Cohort Studies, an Update of the Evidence of the WCRF-AICR Continuous Update Project. Ann. Oncol..

[13] Rosato V., Bosetti C., Levi F., Polesel J., Zucchetto A., Negri E., La Vecchia C. (2013). Risk Factors for Young-Onset Colorectal Cancer. Cancer Causes Control.

[14] Ma Y., Zhang P., Wang F., Yang J., Liu Z., Qin H. (2011). Association between Vitamin D and Risk of Colorectal Cancer: A Systematic Review of Prospective Studies. J. Clin. Oncol..

[15] Wolin K.Y., Yan Y., Colditz G.A., Lee I.M. (2009). Physical Activity and Colon Cancer Prevention: A Meta-Analysis. Br. J. Cancer.

[16] Rostom A., Dubé C., Lewin G., Tsertsvadze A., Barrowman N., Code C., Sampson M., Moher D. (2007). Nonsteroidal Anti-Inflammatory Drugs and Cyclooxygenase-2 Inhibitors for Primary Prevention of Colorectal Cancer: A Systematic Review Prepared for the U.S. Preventive Services Task Force. Ann. Intern. Med..

[17] McNabb S., Harrison T.A., Albanes D., Berndt S.I., Brenner H., Caan B.J., Campbell P.T., Cao Y., Chang-Claude J., Chan A. (2020). Meta-Analysis of 16 Studies of the Association of Alcohol with Colorectal Cancer. Int. J. Cancer.

[18] Botteri E., Iodice S., Bagnardi V., Raimondi S., Lowenfels A.B., Maisonneuve P. (2008). Smoking and Colorectal Cancer: A Meta-Analysis. JAMA.

[19] Kumar A., Gautam V., Sandhu A., Rawat K., Sharma A., Saha L. (2023). Current and Emerging Therapeutic Approaches for Colorectal Cancer: A Comprehensive Review. World J. Gastrointest. Surg..

[20] Schuell B., Gruenberger T., Kornek G.V., Dworan N., Depisch D., Lang F., Schneeweiss B., Scheithauer W. (2005). Side Effects during Chemotherapy Predict Tumour Response in Advanced Colorectal Cancer. Br. J. Cancer.

[21] Zhou J., Ji Q., Li Q. (2021). Resistance to Anti-EGFR Therapies in Metastatic Colorectal Cancer: Underlying Mechanisms and Reversal Strategies. J. Exp. Clin. Cancer Res..

[22] Ding S., Hu C., Fang J., Liu G. (2020). The Protective Role of Probiotics against Colorectal Cancer. Oxid. Med. Cell. Longev..

[23] Liu J., Guo B. (2020). RNA-Based Therapeutics for Colorectal Cancer: Updates and Future Directions. Pharmacol. Res..

[24] Fukuhara H., Ino Y., Todo T. (2016). Oncolytic Virus Therapy: A New Era of Cancer Treatment at Dawn. Cancer Sci..

[25] Rajamanickam S., Agarwal R. (2008). Natural Products and Colon Cancer: Current Status and Future Prospects. Drug Dev. Res..

[26] Filippini T., Malavolti M., Borrelli F., Izzo A.A., Fairweather-Tait S.J., Horneber M., Vinceti M. (2020). Green Tea (Camellia Sinensis) for the Prevention of Cancer. Cochrane Database Syst. Rev..

[27] Botten D., Fugallo G., Fraternali F., Molteni C. (2015). Structural Properties of Green Tea Catechins. J. Phys. Chem. B.

[28] Jigisha A., Nishant R., Navin K., Pankaj G. (2012). Green Tea: A Magical Herb with Miraculous Outcomes. Int. Res. J. Pharm..

[29] Yanagimoto K., Ochi H., Lee K.G., Shibamoto T. (2003). Antioxidative Activities of Volatile Extracts from Green Tea, Oolong Tea, and Black Tea. J. Agric. Food Chem..

[30] Alam M., Ali S., Ashraf G.M., Bilgrami A.L., Yadav D.K., Hassan M.I. (2022). Epigallocatechin 3-Gallate: From Green Tea to Cancer Therapeutics. Food Chem..

[31] Almatrood S.A., Almatroudi A., Khan A.A., Alhumaydh F.A., Alsahl M.A., Rahmani A.H. (2020). Potential Therapeutic Targets of Epigallocatechin Gallate (EGCG), the Most Abundant Catechin in Green Tea, and Its Role in the Therapy of Various Types of Cancer. Molecules.

[32] Zhao T., Li C., Wang S., Song X. (2022). Green Tea (Camellia Sinensis): A Review of Its Phytochemistry, Pharmacology, and Toxicology. Molecules.

[33] Ríos J.L., Francini F., Schinella G.R. (2015). Natural Products for the Treatment of Type 2 Diabetes Mellitus. Planta Med..

[34] Yoneda Y., Kuramoto N., Kawada K. (2019). The Role of Glutamine in Neurogenesis Promoted by the Green Tea Amino Acid Theanine in Neural Progenitor Cells for Brain Health. Neurochem. Int..

[35] Shirakami Y., Shimizu M. (2018). Possible Mechanisms of Green Tea and Its Constituents against Cancer. Molecules.

[36] Lambert J.D., Elias R.J. (2010). The Antioxidant and Pro-Oxidant Activities of Green Tea Polyphenols: A Role in Cancer Prevention. Arch. Biochem. Biophys..

[37] Teramoto M., Yamagishi K., Muraki I., Tamakoshi A., Iso H. (2023). Coffee and Green Tea Consumption and Cardiovascular Disease Mortality Among People with and Without Hypertension. J. Am. Heart Assoc..

[38] Ahmad N., Mukhtar H. (1999). Green Tea Polyphenols and Cancer: Biologic Mechanisms and Practical Implications. Nutr. Rev..

[39] Graham H.N. (1992). Green Tea Composition, Consumption, and Polyphenol Chemistry. Prev. Med..

[40] Tallei T.E., Fatimawali, Niode N.J., Idroes R., Zidan B.M.R.M., Mitra S., Celik I., Nainu F., Aǧagündüz D., Emran T.B. (2021). A Comprehensive Review of the Potential Use of Green Tea Polyphenols in the Management of COVID-19. Evid.-Based Complement. Altern. Med..

[41] Farhan M. (2022). Green Tea Catechins: Nature’s Way of Preventing and Treating Cancer. Int. J. Mol. Sci..

[42] Brimson J.M., Prasanth M.I., Malar D.S., Sharika R., Sivamaruthi B.S., Kesika P., Chaiyasut C., Tencomnao T., Prasansuklab A. (2021). Role of Herbal Teas in Regulating Cellular Homeostasis and Autophagy and Their Implications in Regulating Overall Health. Nutrients.

[43] Li F., Wang Y., Li D., Chen Y., Qiao X., Fardous R., Lewandowski A., Liu J., Chan T.H., Dou Q.P. (2018). Perspectives on the Recent Developments with Green Tea Polyphenols in Drug Discovery. Expert Opin. Drug Discov..

[44] Musial C., Kuban-Jankowska A., Gorska-Ponikowska M. (2020). Beneficial Properties of Green Tea Catechins. Int. J. Mol. Sci..

[45] Del Rio D., Stewart A.J., Mullen W., Burns J., Lean M.E.J., Brighenti F., Crozier A. (2004). HPLC-MSn Analysis of Phenolic Compounds and Purine Alkaloids in Green and Black Tea. J. Agric. Food Chem..

[46] Khan N., Mukhtar H. (2019). Tea Polyphenols in Promotion of Human Health. Nutrients.

[47] Luo Q., Zhang J.R., Li H.B., Wu D.T., Geng F., Corke H., Wei X.L., Gan R.Y. (2020). Green Extraction of Antioxidant Polyphenols from Green Tea (Camellia Sinensis). Antioxidants.

[48] Yokozawa T., Dong E. (1997). Influence of Green Tea and Its Three Major Components upon Low-Density Lipoprotein Oxidation. Exp. Toxicol. Pathol..

[49] Hamilton-Miller J.M.T. (2001). Anti-Cariogenic Properties of Tea (Camellia Sinensis). J. Med. Microbiol..

[50] Chacko S.M., Thambi P.T., Kuttan R., Nishigaki I. (2010). Beneficial Effects of Green Tea: A Literature Review. Chin. Med..

[51] Oh J.W., Muthu M., Pushparaj S.S.C., Gopal J. (2023). Anticancer Therapeutic Effects of Green Tea Catechins (GTCs) When Integrated with Antioxidant Natural Components. Molecules.

[52] Chatterjee P., Chandra S., Dey P., Bhattacharya S. (2012). Evaluation of Anti-Inflammatory Effects of Green Tea and Black Tea: A Comparative in Vitro Study. J. Adv. Pharm. Technol. Res..

[53] Unno K., Pervin M., Nakagawa A., Iguchi K., Hara A., Takagaki A., Nanjo F., Minami A., Nakamura Y. (2017). Blood–Brain Barrier Permeability of Green Tea Catechin Metabolites and Their Neuritogenic Activity in Human Neuroblastoma SH-SY5Y Cells. Mol. Nutr. Food Res..

[54] Prasanth M.I., Sivamaruthi B.S., Chaiyasut C., Tencomnao T. (2019). A Review of the Role of Green Tea (Camellia Sinensis) in Antiphotoaging, Stress Resistance, Neuroprotection, and Autophagy. Nutrients.

[55] Wolfram S. (2007). Effects of Green Tea and Egcg on Cardiovascular and Metabolic Health. J. Am. Coll. Nutr..

[56] Cheng Z., Zhang Z., Han Y., Wang J., Wang Y., Chen X., Shao Y., Cheng Y., Zhou W., Lu X. (2020). A Review on Anti-Cancer Effect of Green Tea Catechins. J. Funct. Foods.

[57] Zhou P., Yu J.F., Zhao C.G., Sui F.X., Teng X., Wu Y. (2013). Bin Therapeutic Potential of EGCG on Acute Renal Damage in a Rat Model of Obstructive Nephropathy. Mol. Med. Rep..

[58] Zhu S., Li Y., Li Z., Ma C., Lou Z., Yokoyama W., Wang H. (2014). Lipase-Catalyzed Synthesis of Acetylated EGCG and Antioxidant Properties of the Acetylated Derivatives. Food Res. Int..

[59] Nakagawa H., Hasumi K., Woo J.T., Nagai K., Wachi M. (2004). Generation of Hydrogen Peroxide Primarily Contributes to the Induction of Fe(II)-Dependent Apoptosis in Jurkat Cells by (−)-Epigallocatechin Gallate. Carcinogenesis.

[60] Shankar S., Suthakar G., Srivastava R.K. (2007). Epigallocatechin-3-Gallate Inhibits Cell Cycle and Induces Apoptosis in Pancreatic Cancer. Front. Biosci..

[61] Cerezo-Guisado M.I., Zur R., Lorenzo M.J., Risco A., Martín-Serrano M.A., Alvarez-Barrientos A., Cuenda A., Centeno F. (2015). Implication of Akt, ERK1/2 and Alternative P38MAPK Signalling Pathways in Human Colon Cancer Cell Apoptosis Induced by Green Tea EGCG. Food Chem. Toxicol..

[62] Walker A., Singh A., Tully E., Woo J., Le A., Nguyen T., Biswal S., Sharma D., Gabrielson E. (2018). Nrf2 Signaling and Autophagy Are Complementary in Protecting Breast Cancer Cells during Glucose Deprivation. Free Radic. Biol. Med..

[63] Enkhbat T., Nishi M., Yoshikawa K., Jun H., Tokunaga T., Takasu C., Kashihara H., Ishikawa D., Tominaga M., Shimada M. (2018). Epigallocatechin-3-Gallate Enhances Radiation Sensitivity in Colorectal Cancer Cells through Nrf2 Activation and Autophagy. Anticancer Res..

[64] Gupta S., Ahmad N., Nieminen A.L., Mukhtar H. (2000). Growth Inhibition, Cell-Cycle Dysregulation, and Induction of Apoptosis by Green Tea Constituent (−)-Epigallocatechin-3-Gallate in Androgen-Sensitive and Androgen-Insensitive Human Prostate Carcinoma Cells. Toxicol. Appl. Pharmacol..

[65] Zan L., Chen Q., Zhang L., Li X. (2019). Epigallocatechin Gallate (EGCG) Suppresses Growth and Tumorigenicity in Breast Cancer Cells by Downregulation of MiR-25. Bioengineered.

[66] Yang C., Du W., Yang D. (2016). Inhibition of Green Tea Polyphenol EGCG((−)-Epigallocatechin-3-Gallate) on the Proliferation of Gastric Cancer Cells by Suppressing Canonical Wnt/β-Catenin Signalling Pathway. Int. J. Food Sci. Nutr..

[67] Li F., Qasim S., Li D., Dou Q.P. (2022). Updated Review on Green Tea Polyphenol Epigallocatechin-3-Gallate as a Cancer Epigenetic Regulator. Semin. Cancer Biol..

[68] Adachi S., Shimizu M., Shirakami Y., Yamauchi J., Natsume H., Matsushima-Nishiwaki R., To S., Weinstein I.B., Moriwaki H., Kozawa O. (2009). (−)-Epigallocatechin Gallate Downregulates EGF Receptor via Phosphorylation at Ser1046/1047 by P38 MAPK in Colon Cancer Cells. Carcinogenesis.

[69] Shimizu M., Shirakami Y., Sakai H., Kubota M., Kochi T., Ideta T., Miyazaki T., Moriwaki H. (2015). Chemopreventive Potential of Green Tea Catechins in Hepatocellular Carcinoma. Int. J. Mol. Sci..

[70] Misra S., Ikbal A.M.A., Bhattacharjee D., Hore M., Mishra S., Karmakar S., Ghosh A., Srinivas R., Das A., Agarwal S. (2022). Validation of Antioxidant, Antiproliferative, and in Vitro Anti-Rheumatoid Arthritis Activities of Epigallo-Catechin-Rich Bioactive Fraction from Camellia Sinensis Var. Assamica, Assam Variety White Tea, and Its Comparative Evaluation with Green Tea Fract. J. Food Biochem..

[71] Yang E.J., Lee J., Lee S.Y., Kim E.K., Moon Y.M., Jung Y.O., Park S.H., Cho M. (2014). La EGCG Attenuates Autoimmune Arthritis by Inhibition of STAT3 and HIF-1α with Th17/Treg Control. PLoS ONE.

[72] Bernatoniene J., Kopustinskiene D.M. (2018). The Role of Catechins in Cellular Responses to Oxidative Stress. Molecules.

[73] Perletti G., Magri V., Vral A., Stamatiou K., Trinchieri A. (2019). Green Tea Catechins for Chemoprevention of Prostate Cancer in Patients with Histologically-Proven HG-PIN or ASAP. Concise Review and Meta-Analysis. Arch. Ital. Urol. Androl..

[74] Sun C.L., Yuan J.M., Koh W.P., Lee H.P., Yu M.C. (2007). Green Tea and Black Tea Consumption in Relation to Colorectal Cancer Risk: The Singapore Chinese Health Study. Carcinogenesis.

[75] Yang G., Zheng W., Xiang Y.B., Gao J., Li H.L., Zhang X., Gao Y.T., Shu X.O. (2011). Green Tea Consumption and Colorectal Cancer Risk: A Report from the Shanghai Men’s Health Study. Carcinogenesis.

[76] Chen Y., Wu Y., Du M., Chu H., Zhu L., Tong N., Zhang Z., Wang M., Gu D., Chen J. (2017). An Inverse Association between Tea Consumption and Colorectal Cancer Risk. Oncotarget.

[77] Ji B.T., Chow W.H., Hsing A.W., Mclaughlin J.K., Dai Q., Gao Y.T., Blot W.J., Fraumeni J.F. (1997). Green Tea Consumption and the Risk of Pancreatic and Colorectal Cancers. Int. J. Cancer.

[78] Luo K.W., Ye W., Li N., Cheng B.H. (2024). Tea Polyphenol EGC Suppresses Colorectal Cancer Cell Proliferation Both in Vitro and in Vivo via Downregulation of STAT3. J. Funct. Foods.

[79] Luo K.W., Xia J., Cheng B.H., Gao H.C., Fu L.W., Luo X. (2021). Le Tea Polyphenol EGCG Inhibited Colorectal-Cancer-Cell Proliferation and Migration via Downregulation of STAT3. Gastroenterol. Rep..

[80] Zhang Z., Zhu Q., Wang S., Shi C. (2023). Epigallocatechin-3-Gallate Inhibits the Formation of Neutrophil Extracellular Traps and Suppresses the Migration and Invasion of Colon Cancer Cells by Regulating STAT3/CXCL8 Pathway. Mol. Cell. Biochem..

[81] Jin H., Gong W., Zhang C., Wang S. (2013). Epigallocatechin Gallate Inhibits the Proliferation of Colorectal Cancer Cells by Regulating Notch Signaling. Onco. Targets. Ther..

[82] Jung Y.D., Kim M.S., Shin B.A., Chay K.O., Ahn B.W., Liu W., Bucana C.D., Gallick G.E., Ellis L.M. (2001). EGCG, a Major Component of Green Tea, Inhibits Tumour Growth by Inhibiting VEGF Induction in Human Colon Carcinoma Cells. Br. J. Cancer.

[83] Chen Y., Wang X.Q., Zhang Q., Zhu J.Y., Li Y., Xie C.F., Li X.T., Wu J.S., Geng S.S., Zhong C.Y. (2017). (−)-Epigallocatechin-3-Gallate Inhibits Colorectal Cancer Stem Cells by Suppressing Wnt/β-Catenin Pathway. Nutrients.

[84] Wubetu G.Y., Shimada M., Morine Y., Ikemoto T., Ishikawa D., Iwahashi S., Yamada S., Saito Y., Arakawa Y., Imura S. (2016). Epigallocatechin Gallate Hinders Human Hepatoma and Colon Cancer Sphere Formation. J. Gastroenterol. Hepatol..

[85] Shimizu M., Deguchi A., Lim J.T.E., Moriwaki H., Kopelovich L., Weinstein I.B. (2005). (−)-Epigallocatechin Gallate and Polyphenon E Inhibit Growth and Activation of the Epidermal Growth Factor Receptor and Human Epidermal Growth Factor Receptor-2 Signaling Pathways in Human Colon Cancer Cells. Clin. Cancer Res..

[86] Oh S., Gwak J., Park S., Yang C.S. (2014). Green Tea Polyphenol EGCG Suppresses Wnt/β-Catenin Signaling by Promoting GSK-3β- and PP2A-Independent β-Catenin Phosphorylation/Degradation. BioFactors.

[87] Morris J., Moseley V.R., Cabang A.B., Coleman K., Wei W., Garrett-Mayer E., Wargovich M.J. (2016). Reduction in Promotor Methylation Utilizing EGCG (Epigallocatechin-3-Gallate) Restores RXRα Expression in Human Colon Cancer Cells. Oncotarget.

[88] Umeda D., Yano S., Yamada K., Tachibana H. (2008). Involvement of 67-KDa Laminin Receptor-Mediated Myosin Phosphatase Activation in Antiproliferative Effect of Epigallocatechin-3-O-Gallate at a Physiological Concentration on Caco-2 Colon Cancer Cells. Biochem. Biophys. Res. Commun..

[89] Lin X., Wang G., Liu P., Han L., Wang T., Chen K., Gao Y. (2021). Gallic Acid Suppresses Colon Cancer Proliferation by Inhibiting SRC and EGFR Phosphorylation. Exp. Ther. Med..

[90] Shimizu M., Shirakami Y., Sakai H., Adachi S., Hata K., Hirose Y., Tsurumi H., Tanaka T., Moriwaki H. (2008). (−)-Epigallocatechin Gallate Suppresses Azoxymethane-Induced Colonic Premalignant Lesions in Male C57BL/KsJ-Db/Db Mice. Cancer Prev. Res..

[91] Shimizu M., Shirakami Y., Sakai H., Yasuda Y., Kubota M., Adachi S., Tsurumi H., Hara Y., Moriwaki H. (2010). (−)-Epigallocatechin Gallate Inhibits Growth and Activation of the VEGF/VEGFR Axis in Human Colorectal Cancer Cells. Chem. Biol. Interact..

[92] Shojaei-Zarghani S., Yari Khosroushahi A., Rafraf M. (2021). Oncopreventive Effects of Theanine and Theobromine on Dimethylhydrazine-Induced Colon Cancer Model. Biomed. Pharmacother..

[93] Adachi S., Nagao T., To S., Joe A.K., Shimizu M., Matsushima-Nishiwaki R., Kozawa O., Moriwaki H., Maxfield F.R., Weinstein I.B. (2008). (−)-Epigallocatechin Gallate Causes Internalization of the Epidermal Growth Factor Receptor in Human Colon Cancer Cells. Carcinogenesis.

[94] Huang Y., Chen Q., Liu Y., Tian R., Yin X., Hao Y., Yang Y., Yang J., Li Z., Yu S. (2023). Association between Tea Consumption and Colorectal Cancer: A Systematic Review and Meta-Analysis of a Population-Based Study. BMC Gastroenterol..

[95] Zhu M.-Z., Lu D.-M., Ouyang J., Zhou F., Huang P.-F., Gu B.-Z., Tang J.-W., Shen F., Li J.-F., Li Y.-L. (2020). Tea Consumption and Colorectal Cancer Risk: A Meta-Analysis of Prospective Cohort Studies. Eur. J. Nutr..

[96] Yu F., Jin Z., Jiang H., Xiang C., Tang J., Li T., He J. (2014). Tea Consumption and the Risk of Five Major Cancers: A Dose-Response Meta-Analysis of Prospective Studies. BMC Cancer.

[97] Wang Z.H., Gao Q.Y., Fang J.Y. (2012). Green Tea and Incidence of Colorectal Cancer: Evidence from Prospective Cohort Studies. Nutr. Cancer.

[98] Zhang X., Albanes D., Beeson W.L., Van Den Brandt P.A., Buring J.E., Flood A., Freudenheim J.L., Giovannucci E.L., Goldbohm R.A., Jaceldo-Siegl K. (2010). Risk of Colon Cancer and Coffee, Tea, and Sugar-Sweetened Soft Drink Intake: Pooled Analysis of Prospective Cohort Studies. J. Natl. Cancer Inst..

[99] Wang X.J., Zeng X.T., Duan X.L., Zeng H.C., Shen R., Zhou P. (2012). Association between Green Tea and Colorectal Cancer Risk: A Meta-Analysis of 13 Case-Control Studies. Asian Pacific J. Cancer Prev..

[100] Wang Z.J., Ohnaka K., Morita M., Toyomura K., Kono S., Ueki T., Tanaka M., Kakeji Y., Maehara Y., Okamura T. (2013). Dietary Polyphenols and Colorectal Cancer Risk: The Fukuoka Colorectal Cancer Study. World J. Gastroenterol..

[101] Wada K., Oba S., Tsuji M., Goto Y., Mizuta F., Koda S., Uji T., Hori A., Tanabashi S., Matsushita S. (2019). Green Tea Intake and Colorectal Cancer Risk in Japan: The Takayama Study. Jpn. J. Clin. Oncol..

[102] Shimizu M., Fukutomi Y., Ninomiya M., Nagura K., Kato T., Araki H., Suganuma M., Fujiki H., Moriwaki H. (2008). Green Tea Extracts for the Prevention of Metachronous Colorectal Adenomas: A Pilot Study. Cancer Epidemiol. Biomark. Prev..

[103] Suzuki Y., Tsubono Y., Nakaya N., Koizumi Y., Suzuki Y., Shibuya D., Tsuji I. (2005). Green Tea and the Risk of Colorectal Cancer: Pooled Analysis of Two Prospective Studies in Japan. J. Epidemiol..

[104] Kim H., Lee J., Oh J.H., Chang H.J., Sohn D.K., Shin A., Kim J. (2019). Protective Effect of Green Tea Consumption on Colorectal Cancer Varies by Lifestyle Factors. Nutrients.

[105] Shin C.M., Lee D.H., Seo A.Y., Lee H.J., Kim S.B., Son W.C., Kim Y.K., Lee S.J., Park S.H., Kim N. (2018). Green Tea Extracts for the Prevention of Metachronous Colorectal Polyps among Patients Who Underwent Endoscopic Removal of Colorectal Adenomas: A Randomized Clinical Trial. Clin. Nutr..

[106] Chen H.Y., Sun Z.J., Li C.H., Chou Y.T., Chang C.J., Lu F.H., Yang Y.C., Wu J.S. (2021). Cumulative Tea Consumption Is Inversely Associated with Colorectal Adenomas in Adults: A Cross-Sectional Study in a Taiwanese Population. Cancer Epidemiol..

[107] Yang G., Shu X.O., Li H., Chow W.H., Ji B.T., Zhang X., Gao Y.T., Zheng W. (2007). Prospective Cohort Study of Green Tea Consumption and Colorectal Cancer Risk in Women. Cancer Epidemiol. Biomark. Prev..

[108] Li X., Yu C., Guo Y., Bian Z., Shen Z., Yang L., Chen Y., Wei Y., Zhang H., Qiu Z. (2019). Association between Tea Consumption and Risk of Cancer: A Prospective Cohort Study of 0.5 Million Chinese Adults. Eur. J. Epidemiol..

[109] Wu Y., Jin M., Liu B., Liang X., Yu Y., Li Q., Ma X., Yao K., Chen K. (2011). The Association of XPC Polymorphisms and Tea Drinking with Colorectal Cancer Risk in a Chinese Population. Mol. Carcinog..

[110] Hua R.X., Zhuo Z.J., Zhu J., Zhang S.D., Xue W.Q., Zhang J.B., Xu H.M., Li X.Z., Zhang P.F., He J. (2016). XPG Gene Polymorphisms Contribute to Colorectal Cancer Susceptibility: A Two-Stage Case-Control Study. J. Cancer.

[111] Yu Y., Zhang M., Pan Y., Jin M., Jiang X., Zhang S., Wu Y., Ni Q., Li Q., Chen K. (2012). PLA2G4A Mutants Modified Protective Effect of Tea Consumption against Colorectal Cancer. Int. J. Color. Dis..

[112] Zamora-Ros R., Cayssials V., Jenab M., Rothwell J.A., Fedirko V., Aleksandrova K., Tjønneland A., Kyrø C., Overvad K., Boutron-Ruault M.C. (2018). Dietary Intake of Total Polyphenol and Polyphenol Classes and the Risk of Colorectal Cancer in the European Prospective Investigation into Cancer and Nutrition (EPIC) Cohort. Eur. J. Epidemiol..

[113] Dik V.K., Bueno-De-Mesquita H.B., Van Oijen M.G.H., Siersema P.D., Uiterwaal C.S.P.M., Van Gils C.H., Van Duijnhoven F.J.B., Cauchi S., Yengo L., Froguel P. (2014). Coffee and Tea Consumption, Genotype-Based CYP1A2 and NAT2 Activity and Colorectal Cancer Risk—Results from the EPIC Cohort Study. Int. J. Cancer.

[114] Seufferlein T., Ettrich T.J., Menzler S., Messmann H., Kleber G., Zipprich A., Frank-Gleich S., Algül H., Metter K., Odemar F. (2022). Green Tea Extract to Prevent Colorectal Adenomas, Results of a Randomized, Placebo-Controlled Clinical Trial. Am. J. Gastroenterol..

[115] Hartman T.J., Tangrea J.A., Pietinen P., Malila N., Virtanen M., Taylor P.R., Albanes D. (1998). Tea and Coffee Consumption and Risk of Colon and Rectal Cancer in Middle-Aged Finnish Men. Nutr. Cancer.

[116] Terry P., Wolk A. (2001). Tea Consumption and the Risk of Colorectal Cancer in Sweden. Nutr. Cancer.

[117] Shi S., Wang K., Zhong R., Cassidy A., Rimm E.B., Nimptsch K., Wu K., Chan A.T., Giovannucci E.L., Ogino S. (2023). Flavonoid Intake and Survival after Diagnosis of Colorectal Cancer: A Prospective Study in 2 US Cohorts. Am. J. Clin. Nutr..

[118] Su L.J., Arab L. (2002). Tea Consumption and the Reduced Risk of Colon Cancer—Results from a National Prospective Cohort Study. Public Health Nutr..

[119] Cerhan J.R., Putnam S.D., Bianchi G.D., Parker A.S., Lynch C.F., Cantor K.P. (2001). Tea Consumption and Risk of Cancer of the Colon and Rectum. Nutr. Cancer.

[120] Green C.J., de Dauwe P., Boyle T., Tabatabaei S.M., Fritschi L., Heyworth J.S. (2014). Tea, Coffee, and Milk Consumption and Colorectal Cancer Risk. J. Epidemiol..

[121] Li N., Taylor L.S., Ferruzzi M.G., Mauer L.J. (2012). Kinetic Study of Catechin Stability: Effects of Ph, Concentration, and Temperature. J. Agric. Food Chem..

[122] Wu Q.Q., Liang Y.F., Ma S.B., Li H., Gao W.Y. (2019). Stability and Stabilization of (–)-Gallocatechin Gallate under Various Experimental Conditions and Analyses of Its Epimerization, Auto-Oxidation, and Degradation by LC-MS. J. Sci. Food Agric..

[123] Murakami A. (2014). Dose-Dependent Functionality and Toxicity of Green Tea Polyphenols in Experimental Rodents. Arch. Biochem. Biophys..

[124] Vieira I.R.S., Tessaro L., Lima A.K.O., Velloso I.P.S., Conte-Junior C.A. (2023). Recent Progress in Nanotechnology Improving the Therapeutic Potential of Polyphenols for Cancer. Nutrients.

[125] Granja A., Frias I., Neves A.R., Pinheiro M., Reis S. (2017). Therapeutic Potential of Epigallocatechin Gallate Nanodelivery Systems. Biomed Res. Int..

[126] Yang Q.Q., Wei X.L., Fang Y.P., Gan R.Y., Wang M., Ge Y.Y., Zhang D., Cheng L.Z., Corke H. (2020). Nanochemoprevention with Therapeutic Benefits: An Updated Review Focused on Epigallocatechin Gallate Delivery. Crit. Rev. Food Sci. Nutr..

[127] Das T., Mondal S., Das S., Das S., Das Saha K. (2024). Enhanced Anticancer Activity of (-)-Epigallocatechin-3-Gallate (EGCG) Encapsulated NPs toward Colon Cancer Cell Lines. Free Radic. Biol. Med..

[128] Borah G., Bharali M.K. (2021). Green Tea Catechins in Combination with Irinotecan Attenuates Tumorigenesis and Treatment-Associated Toxicity in an Inflammation-Associated Colon Cancer Mice Model. J. Egypt. Natl. Canc. Inst..

[129] Xu G., Ren G., Xu X., Yuan H., Wang Z., Kang L., Yu W., Tian K. (2010). Combination of Curcumin and Green Tea Catechins Prevents Dimethylhydrazine-Induced Colon Carcinogenesis. Food Chem. Toxicol..

